# Combining Biologically Active β-Lactams Integrin Agonists with Poly(l-lactic acid) Nanofibers: Enhancement of Human Mesenchymal Stem Cell Adhesion

**DOI:** 10.1021/acs.biomac.9b01550

**Published:** 2020-02-03

**Authors:** Giulia Martelli, Nora Bloise, Andrea Merlettini, Giovanna Bruni, Livia Visai, Maria Letizia Focarete, Daria Giacomini

**Affiliations:** † Department of Chemistry “Giacomo Ciamician”, University of Bologna, Via Selmi 2, 40126 Bologna, Italy; ‡ Department of Molecular Medicine (DMM), Biochemistry Unit, Center for Health Technologies (CHT), UdR INSTM University of Pavia, Viale Taramelli 3/B, 27100 Pavia, Italy; ∥ Department of Occupational Medicine, Toxicology and Environmental Risks, Istituti Clinici Scientifici Maugeri S.p.A, IRCCS, Via S. Boezio 28, 27100 Pavia, Italy; § Department of Chemistry, Section of Physical Chemistry, University of Pavia, Viale Taramelli 16, 27100 Pavia, Italy

## Abstract

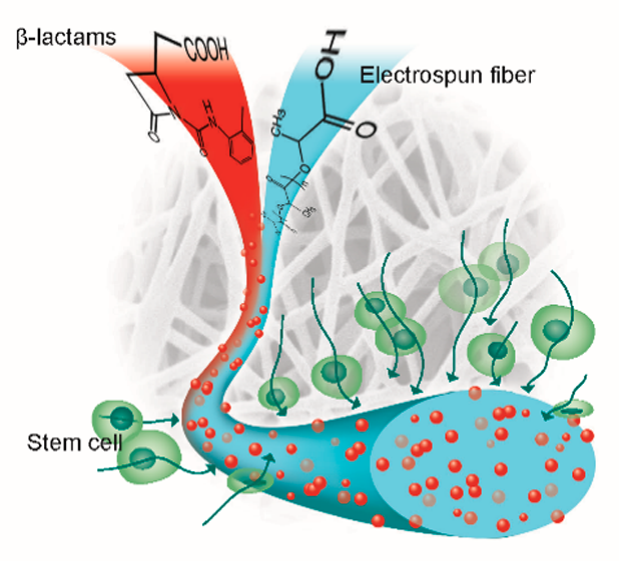

Regulating stem cell adhesion and growth onto functionalized biomaterial scaffolds is an important issue in the field of tissue engineering and regenerative medicine. In this study, new electrospun scaffolds of poly(l-lactic acid) (PLLA), as bioresorbable polymer, and β-lactam compounds agonists of selected integrins, as functional components with cell adhesive properties, are designed. The new β-lactam-PLLA scaffolds contribute significantly in guiding protein translation involved in human bone marrow mesenchymal stem cells (hBM-MSC) adhesion and integrin gene expression. Scanning electron microscopy, confocal laser scanning microscopy, and Western Blot analyses reveal that GM18-PLLA shows the best results, promoting cell adhesion by significantly driving changes in focal adhesion proteins distribution (β_1_ integrin and vinculin) and activation (pFAK), with a notable increase of GM18-targets subunits integrin gene expression, α_4_ and β_1_. These novel functionalized submicrometric fibrous scaffolds demonstrate, for the first time, the powerful combination of selective β-lactams agonists of integrins with biomimetic scaffolds, suggesting a designed rule that could be suitably applied to tissue repair and regeneration.

## Introduction

Biomaterials that are able to instruct cell responses through a control of the cell adhesion pathway activation play a crucial role in tissue engineering and have been thoroughly investigated in several studies.^1^ Unlike natural polymers, synthetic polymeric biomaterials used in tissue engineering applications lack biological activity and typically do not promote excellent cell adhesion and growth. Therefore, scaffold functionalization with growth factors, adhesion peptides, and cytokines has been receiving considerable attention since it plays an important role in the communication and information transfer between the cells and their microenvironment.^2^ The control of cell adhesion, so that particular signaling pathways would be enhanced or suppressed, can be achieved through bioactive scaffolds that are able to engage cells through specific integrins.^3^ Integrins are a family of cell adhesion receptors^4^ constituted of two independent subunits, alpha (α) and beta (β), which in mammals assemble into 24 heterodimeric pairs each with peculiar functions and tissue specificity. Integrins are not just adhesion receptors that mediate dynamic adhesive cell–cell and cell–matrix interactions, but they can transmit information into cells to regulate migration, survival, and growth. The activation of intracellular signaling pathways, called outside-in signaling, occurs upon the binding of specific ligands in the extracellular domain of the integrins.^5a^ The outside-in signaling, in turn, triggers a vast array of intracellular signaling events that control cell shape, motility, proliferation, and cell-type-specific gene expression.^5b^ Several studies were devoted to finding non-natural ligands which inhibit integrin function (antagonists), and some preclinical studies suggested that integrin antagonists might be useful to suppress tumor angiogenesis and growth.^6^ Less attention was addressed to those ligands that promote integrin activation, but it was recently found that integrin agonists could open novel opportunities for therapeutics, which gain benefits in increasing rather than decreasing integrin-dependent adhesion.^7^

Recently, a novel series of monocyclic β-lactam derivatives was designed and synthesized by a structure-based strategy to target RGD-binding and leukocyte integrins.^8^ From a biological standpoint, the β-lactam ring is considered to be a privileged structure because of its peculiar heterocyclic framework able to provide ligands with different pharmacological profiles.^9^

The chemical structure of the new integrin ligands was designed with an amine, a carboxylate side chain, and the β-lactam ring as a site of conformational restriction to provide a favorable alignment on the receptor to satisfy the crucial requirements for integrin affinity and selectivity. The library of β-lactam derivatives was evaluated by investigating the effects on integrin-mediated cell adhesion and signaling in cell lines overexpressing integrins α_v_β_3_, α_v_β_5_, α_v_β_6_, α_5_β_1_, α_IIb_β_3_, α_4_β_1_, and α_L_β_2_.^8a^ Among the new compounds, potent agonists that could induce cell adhesion and promote cell signaling mediated by integrins α_v_β_3_, α_v_β_5_, α_5_β_1_, or α_4_β_1_ were successfully obtained.^8^

To stimulate cell adhesion on biomaterials, some adhesive peptides that contain the RGD tripeptide were used.^10^ However, it would be important to consider that the RGD sequence is recognized by different integrin classes, so the specificity of cell activation could be highly limited.^11^ On the contrary, the use of the new β-lactam integrin agonists could provide the possibility to generate new functional biomaterials with targeted cell specificity because of the integrin selectivity exerted by the new ligands.

Electrospinning is a powerful technology to fabricate nanofibrous scaffolds.^12^ The great potential of electrospun systems is mainly expressed in the biomedical field where they are employed for tissue engineering applications, drug delivery systems, diagnostics, and as biosensors.^13^ Most of the functionalization approaches of electrospun scaffolds with biomolecules—such as growth factor, nucleic acids, cell adhesive peptides, therapeutic molecules, bioprobes, and integrin-binding ligands—are related to surface modification.^14^ However, biomolecules can also be incorporated into the bulk fiber material directly during the fabrication process. This approach allows greater amounts of biomolecule incorporation and shows improved bioactivity if compared to surface modification techniques.^14c,15^ Furthermore, when biomolecules are embedded into the bulk material of the fibers, it is possible to have their release in the surrounding medium by diffusion or by degradation in the case of a biodegradable material, with a release that usually involves an initial burst followed by a steady-state release.^14b^

**Figure 1:**
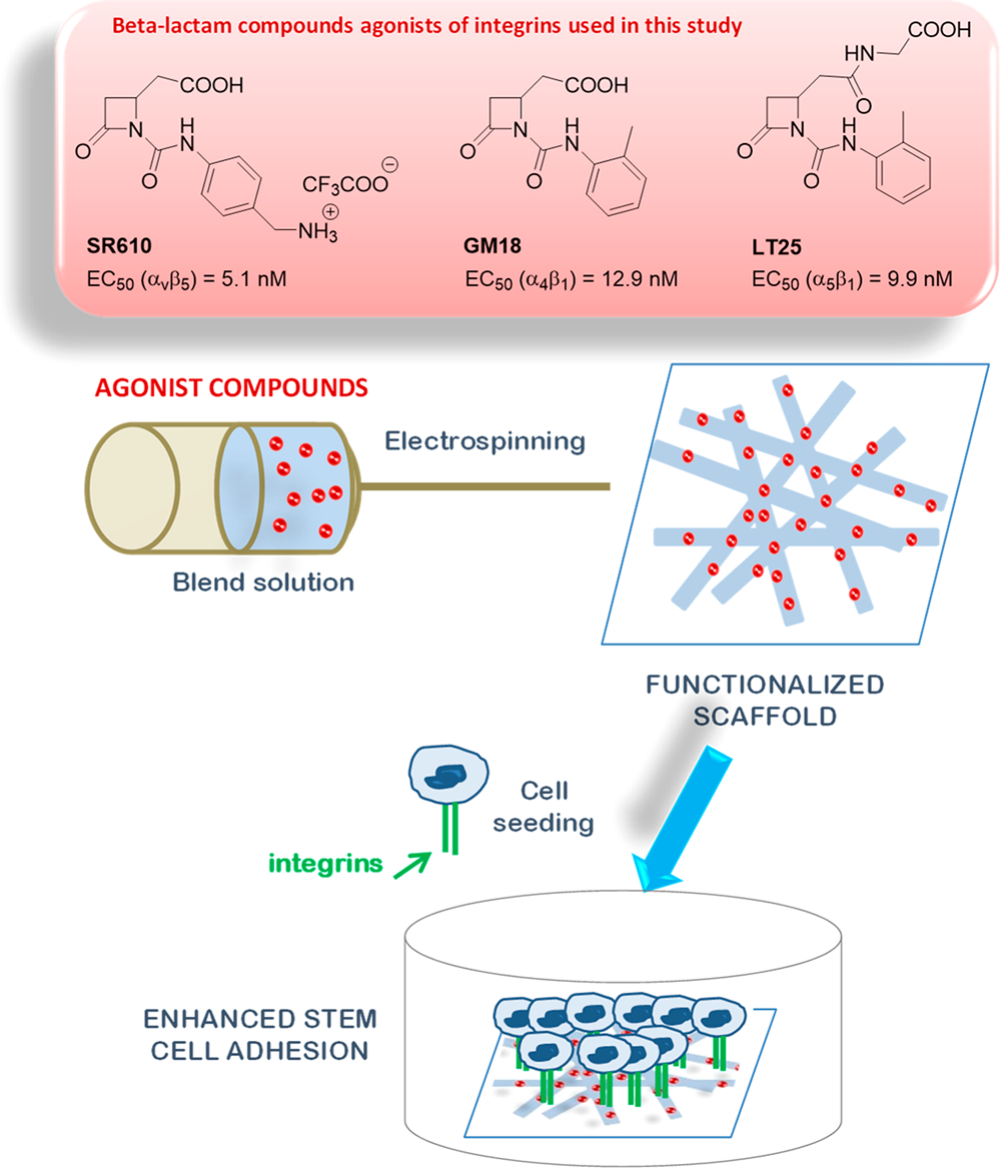
Preparation of functionalized scaffolds by incorporating β-lactam compounds into poly(l-lactic acid) (PLLA) nanofibers obtained by electrospinning technique. The new scaffolds were then used in stem cell experiments. EC_50_ values of β-lactams activity as agonists of specific integrins are reported.

Despite the large number of studies reporting the use of electrospun fibers incorporating bioactive molecules as scaffolds for tissue engineering and drug delivery applications, scaffold functionalization with β-lactam-based integrin agonists has never been reported. Accordingly, in this study the functionalization of electrospun PLLA fibers with novel β-lactam derivatives was explored for the first time to produce a new biologically active tridimensional scaffold that would enhance adhesion and promote the intracellular signaling of human bone marrow-derived mesenchymal stem cells, a clinically relevant cell type for regenerative medicine and tissue engineering therapies (Figure 1).^16^

## Experimental Section

### Materials

Poly(l-lactic acid), PLLA (Lacea H.100-E, Mw = 8.4 × 10^4^ g/mol, PDI = 1.7), was supplied by Mitsui Fine Chemicals (Dusseldorf, Germany). Dichloromethane (DCM) and dimethylformamide (DMF) were purchased from Sigma-Aldrich and used without further purification. β-Lactam SR610, GM18, and LT25 were prepared accordingly to the multistep synthesis described in ref (8).

### Functionalized Scaffold Fabrication

The homemade electrospinning apparatus consisted of a high-voltage power supply (Spellman SL 50 P 10/CE/230), a syringe pump (KD Scientific 200 series), a glass syringe containing the polymer solution and connected to a stainless steel blunt-ended needle (inner diameter = 0.51 mm) through a PTFE tube. A rotating collector (length = 120 mm, diameter = 50 mm, 1000 rpm) was used to produce mats made of fibers randomly oriented. Electrospinning was performed at room temperature (RT) and relative humidity 50–60%. Blends of the polymer and the β-lactam compounds were prepared by dissolving the two components in a mixed solvent of DCM:DMF = 65:35 v/v at a polymer concentration of 13% w/v and a concentration of β-lactam of 10 wt % with respect to the polymer. A PLLA solution at the same polymer concentration in the same solvent mixture was also prepared. The polymeric solutions were electrospun by applying the following processing conditions: applied voltage = 22 kV, feed rate = 1 mL/h, needle-to-collector distance = 15 cm.

### Quantification of the β-Lactams in the Fibers

The amount of β-lactams SR610, GM18, and LT25 loaded on PLLA mats was quantitatively assessed by complete dissolution of weighted mat samples (1–3 mg) in CH_2_Cl_2_ (1 mL), evaporation under reduced pressure, and extraction with methanol (2 mL). The methanol solutions were analyzed in triplicate by HPLC-UV; three independent experiments were carried out for each β-lactam-PLLA. Linear calibration curves for the HPLC-UV analysis of β-lactams in supernatant solutions were established at 254 nm.

### Release Studies

The *in vitro* release profile of β-lactams from the corresponding functionalized PLLA electrospun nanofibers—SR610-PLLA (6.18% of loaded β-lactam), GM18-PLLA (7.48%), and LT25-PLLA (6.31%) was investigated by HPLC analysis.

In a 10 mL test tube a β-lactam-PLLA mat (1–3 mg) was suspended in 0.5 mL of phosphate buffered solution (PBS, 0.1 M, pH 7.4). Experiments in triplicate were conducted at 37 °C in thermostat with sampling and refresh of PBS at set time intervals (see Figures 3A,B and S5) to allow a constant new release of the molecules, according to what was reported in ref (17). At each time point, the supernatant was separated and the released concentration of the β-lactam was determined by HPLC-UV analysis. The PLLA mat was incubated again with a fresh solution of the medium (0.5 mL). The prewetting treatment consisted of dipping the β-lactam-PLLA mat in (1) 0.5 mL of phosphate buffer (PBS, 0.1 M, pH 7.4)/ethanol 70:30 solution (2 s), (2) 0.5 mL of PBS (5 min), and (3) 0.5 mL of PBS (5 min). The prewetted mat was then used for the release study as described above. The amount of β-lactam released during the washing phase was determined by HPLC-UV analysis. Linear calibration curves for the HPLC-UV analysis of β-lactams in supernatant solutions were established at 254 nm. For GM18-PLLA the release study was established also in Milli-Q water and acetate buffer pH = 5, 0.1 M following the procedure reported above.

### Characterization Methods

ATR-FTIR spectra were recorded on an Alpha FT IR Bruker spectrometer with platinum ATR single reflection diamond module. As a reference, the background spectrum of air was collected before the acquisition of each sample spectrum. Spectra were recorded with a resolution of 4 cm^–1^ and 32 scans were averaged for each spectrum (scan range 4000–450 cm^–1^). HPLC-MS analyses were performed with an Agilent Technologies HP1100 instrument, equipped with a ZOBRAX-Eclipse XDΒ-C8 Agilent Technologies column, mobile phase: H_2_O/CH_3_CN, 0.4 mL/min, gradient from 30% to 80% of CH_3_CN in 8 min, 80% of CH_3_CN until 25 min, coupled with an Agilent Technologies MSD1100 single-quadrupole mass spectrometer, full scan mode from *m*/*z* = 50 to 2600, scan time 0.1 s in positive ion mode, ESI spray voltage 4500 V, nitrogen gas 35 psi, drying gas flow 11.5 mL/min, fragmentor voltage 20 V. ^1^H and ^13^C NMR spectra were recorded with an INOVA 400 instrument with a 5 mm probe, CDCl_3_ or d-4 methanol solutions. Scanning electron microscopy (SEM) observations were carried out using a Philips 515 SEM at an accelerating voltage of 15 kV, on samples sputter-coated with gold. The distribution of fiber diameters (average and standard deviation) was measured on the SEM images of about 200 fibers by means of acquisition and image analysis software (EDAX Genesis). Thermogravimetric analysis (TGA) measurements were performed with a TA Instruments Q500 thermogravimetric analyzer from room temperature to 600 °C at a heating rate of 10 °C/min in a nitrogen atmosphere. Differential scanning calorimeter (DSC) measurements were carried out by using a TA Instruments Q2000 apparatus. About 5 mg of sample was placed in Tzero aluminum pans and subjected to a heating scan at 20 °C/min from −90 °C to +200 °C, quenched to −90 °C, and then heated up to 200 °C at 20 °C/min under nitrogen atmosphere.

### Cell Culture

hBM-MSCs were isolated and phenotypically analyzed to assess their mesenchymal properties according to the International Society for Cellular Therapy as previously described.^18^ The study protocols were approved by the Institutional Review Board of the Fondazione IRCCS Policlinico San Matteo and the University of Pavia (2011). Written informed consent was obtained from all the participants involved in this study. The cells used in all experiments were mainly at passage 4–5. hBM-MSCs were cultured at 37 °C in a humidified incubator with 5% CO_2_ in maintenance medium, low-glucose DMEM (Dulbecco’s modified Eagle’s medium) supplemented with 10% Mesencult, 2% glutamine, 1% penicillin–streptomycin (P–S), and 1% amphotericin B (Lonza Group Ltd.).

### Cell Seeding Conditions

Prior to cell seeding, all scaffolds were shaped into suitably sized pieces, assembled with CellCrown support for 24-well plates (Scaffdex, Tampere, Finland) and then sterilized using γ radiation. Unlike unloaded agonist experiments reported in Figure S9 where fibronectin-coated wells were used as positive controls, in the biological experiments including all the β-lactam-functionalized PLLA scaffolds, hBM-MSCs cultured on tissue culture plates (TCPS) were chosen as positive controls (Figures S7 and S10).

### Cell Adhesion Studies

To ensure a maximum number of attached cells for scaffolds, a cell suspension of 1 × 10^5^ cells was added onto the top of each scaffold and incubated at 37 °C in humidified atmosphere with 5% CO_2_. After 2 h, cell-seeded scaffolds were washed with 1× PBS and subsequently analyzed in terms of viability, morphology, and qualitative/quantitative analysis of specific proteins involved in the adhesion process.

### Cell Proliferation Studies

A drop of cell suspension (1.0 × 10^5^ cells) was added onto the top of the wetted plain PLLA and agonists-PLLA scaffolds and, after 0.5 h, 1 mL of culture medium was added to cover the scaffolds. The culture medium was changed every 3 days. After 3 and 7 days of incubation cell viability, morphology and gene expression were evaluated.

### Cell Viability

A 3-(4,5-dimethylthiazole-2-yl)-2,5-diphenyl tetrazolium bromide-based assay (MTT; Sigma-Aldrich) was used to estimate the number of viable cells on TCPS, plain PLLA, and agonists-PLLA scaffolds as described in ref (19). A standard curve of cell viability was used to express the results as percentage viable cells in comparison with the initial state (day 0 = T_0_).

### Scanning Electron Microscopy (SEM) Observation

Cells were seeded on the different agonist-PLLA scaffolds and plastic cell culture coverslip disks (as positive control, Thermanox Plastic, Nalge Nunc International, New York, NY), and then treated as previously described.^20^ The specimens were gold sputter-coated under nitrogen and observed at 500×, 1500×, and 5000× magnification, respectively, using a Leica Cambridge Stereoscan 440 microscope (Leica Microsystems, Bensheim, Germany).

### Confocal Laser Scanning Microscopy (CLSM) Analysis

After 2 h of culture, cell-seeded onto TCPS, plain PLLA, and agonist-PLLA scaffolds were washed with PBS, fixed with 4% (w/v) paraformaldehyde solution (PFA) for 30 min at 4 °C, and permeabilized with 0.1% Triton X-100 for 5 min. In order to visualize the F-actin cytoskeleton organization, cells were stained with Tetramethylrhodamine B isothiocyanate (TRITC) phalloidin conjugate solution (10 μg/mL, EX/EM maxima ∼540/575, Sigma-Aldrich) in PBS for 40 min at RT. For focal adhesion detection, cells were incubated with primary mouse anti-α-vinculin antibody (1:500 in 1% bovine serum albumin, BSA, BosterBio, Pleasanton, CA, USA), anti-β_1_-integrin (1:100 in 1% BSA, NSJ Bioreagents, San Diego, CA, USA), or anti-p-FAK (pY397, 1:250, Santa Cruz, USA). Afterward, samples were incubated with specific secondary antibodies for immunofluorescence, all used at a concentration of 1:1.500 in 1% BSA. Hoechst 33342 (2 μg/mL) was used for nuclei staining. The images were taken using a TCS SPII confocal microscope (Leica Microsystems, Bensheim, Germany) equipped with a digital image capture system at 20× and 40× magnification. Orthogonal views of stack images were also taken (40× magnification, Figures S7C and S10). The quantitative data were derived from analysis of 5 fields per image, and a total of three images were analyzed for each experiment. ImageJ software was used to quantify fluorescent intensity expressed as corrected total cell fluorescence (CTCF) (CTFC = integrated density – (area of selected cell × mean fluorescence of background readings)) according to previous studies.^21^

### Western Blot

Cells were scraped from all samples, including TCPS, and lysed with ice-cold lysis buffer (RIPA buffer 1× containing 1 mM and 1× protease inhibitor (Protease Inhibitor Tablets, SIGMA) for 30 min on ice. The lysates were then used for Western blot analysis according to a literature protocol.^22^ Primary antibodies anti-vinculin (diluted 1:250), anti-β_1_-integrin (diluted 1:1000), anti-FAK (diluted 1:1000), anti-phosphorylated FAK (pY397) (diluted 1:1000), anti-β-actin (diluted 1:500), and appropriate secondary HRP-conjugated antibodies were used. Detection was performed with Western Chemiluminescent HRP substrate, (LI-COR) and revealed using an ImageQuant LAS4000 imaging system (GE Healthcare). Band densitometry analysis was carried out with ImageJ software.

### Real-Time qPCR

At days 3 and 7 of culture, the total RNA was extracted from cells seeded on prewetted GM18-PLLA and PLLA using Nucleozol reagent, according to the manufacturer’s protocol (Macherey-Nagel, Düren, Germany). The reverse transcription was performed using the iScript cDNA Synthesis Kit (Thermo Fisher Scientific, Waltham, Massachusetts, USA). Quantitative reverse-transcription polymerase chain reaction (qRT-PCR) analysis was performed in a 96-well optical reaction plate using a qPCR Quant3 Studio (Applied BioSystem, Foster City, CA, USA). Reactions were performed in 10 μL with 4 μL of cDNA, 5 μL Brilliant SYBER Green qPCR Master Mix (Bio-Rad Laboratories), 0.1 μL of each primer, and 7.2 μL H_2_O. The PCR conditions were as follows: 3 min at 95 °C, 40 cycles of 5 s at 95 °C, and 23 s at 60 °C. The reaction mixture without cDNA was used as a negative control in each run. Gene expression was analyzed in triplicate and normalized to the CT mean of GAPDH housekeeping gene expression using the ΔΔ*C*_t_ Livak method. The graphs show the fold increase of gene expression related to cells at the initial state (day 0).^23^ Integrin primers used are listed in Table S3.

### Statistics and Data Analysis

Each experiment reported in the Results section was run in triplicate, at least in three separate experiments. Results were expressed as the mean ± standard deviation. Statistical analysis was performed by one-way variance analysis (ANOVA), followed by post hoc Bonferroni test for multiple comparisons (significance level of *p* ≤ 0.05). All calculations were generated using GraphPad (GraphPad Inc., San Diego, CA, USA).

## Results and Discussion

### Functionalized Scaffold Fabrication and Characterization

One of the main challenges in tissue engineering is to obtain a scaffold that can induce cell adhesion and promote cell signaling mediated by specific integrin classes. A possible strategy to achieve this goal is scaffold functionalization with suitable integrin agonists, as proposed in the present work. A scheme representing the preparation of functionalized scaffolds to be tested with mesenchymal stem cells is shown in Figure 1.

PLLA was selected as the bioresorbable polymeric matrix, to create scaffolds made of electrospun submicrometric fibers. Among the library of β-lactams recently developed,^8^ some derivatives were found to strongly promote cell adhesion mediated by integrins at a nanomolar level. According to this process, the most active compounds were selected as candidates for loading in PLLA electrospun nanofibers.

Three β-lactams, SR610, GM18, and LT25, were chosen for their structural variability and their specific selectivity toward different integrin classes in enhancing cell adhesion; the preferential integrin class selectivity and the corresponding EC_50_ values in cell-adhesion assays^8^ are reported in Figure 1. Compounds SR610, GM18, and LT25 were obtained with a multistep synthesis (Supporting Information Figure S1) in satisfactory yields and good purity (HPLC-MS assays >95%) as previously described.^8^

**Figure 2:**
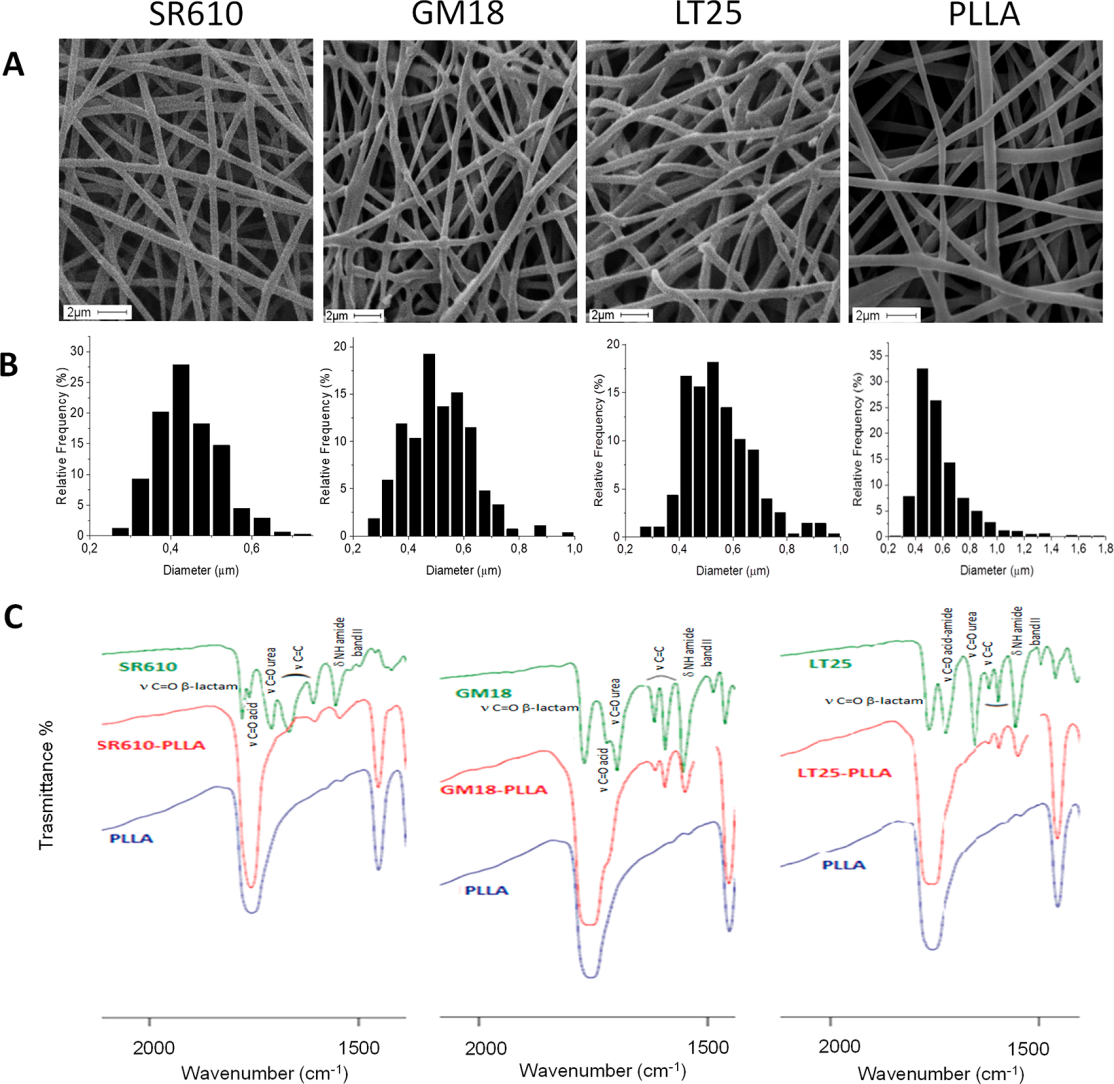
(A) SEM images of SR610-PLLA (6.18 wt %), GM18-PLLA (7.48 wt %), and LT25-PLLA (6.31 wt %) samples, and plain PLLA; and (B) the corresponding fiber dimensional analysis. (C) ATR-FTIR analysis of the functionalized fibers (red lines) SR610-PLLA (left), GM18-PLLA (center), and LT25-PLLA (right), in comparison with pure compounds (green lines) and PLLA alone (blue line). Enlarged and selected parts of ATR-FTIR spectra are reported. Assignments of the main bands are indicated. The entire IR spectra for all samples are reported in Figure S2.

The loading of a 10 wt % of the β-lactams SR610, GM18, and LT25 into PLLA nanofibers was conducted by electrospinning blend solutions of the two components in a common solvent by using processing conditions previously optimized for PLLA.^24^ Plain PLLA fibers were also obtained for comparison. Scaffolds made of uniform, bead-free, and randomly arranged fibers were obtained (scanning electron microscopy (SEM) analysis in Figure 2). Fiber diameter distribution shows that fibers with an average diameter around 500 nm were obtained for all samples (Table S1) with a fiber distribution slightly broader for the functionalized fibers with respect to plain PLLA (Figure 2). Morphological analysis demonstrated that the addition of a nominal amount of 10 wt % of β-lactam derivatives did not significantly modify the PLLA solution properties. The physical–chemical properties of the electrospun β-lactam-PLLA fibers were also evaluated through attenuated total reflectance infrared spectroscopy (ATR FTIR) and compared to those of untreated fibers. Figure 2 reports ATR-FTIR spectra in the region 2100–1480 cm^–1^ of β-lactam-PLLA samples compared to pure SR610, GM18, LT25, and plain PLLA (the entire IR spectra for all samples are reported in Figure S2). In the spectra of the molecules alone, it was possible to identify the typical IR bands of β-lactams with the relative assignments. In the β-lactams-PLLA spectra, the strong C=O stretching absorption of the polymer at 1756 cm^–1^ completely overlapped the characteristic bands of the β-lactam C=O groups. Nevertheless, the bands of aromatic C=C stretching and of secondary amide NH bending at around 1600 and 1555 cm^–1^ clearly appeared in the β-lactam-PLLA scaffolds and confirmed the presence of the molecules in the functionalized nanofibers. Moreover, these bands show the same frequencies of the pure molecules, thus attesting to the molecular integrity of the β-lactams upon loading.

In addition, PLLA was not affected by the presence of the β-lactam compounds, since compared to plain PLLA, no shift of infrared bands was detected in the functionalized nanofibers.

The amount of β-lactams loaded on the functionalized PLLA mats was quantitatively assessed by complete dissolution of weighted samples of the scaffolds in CH_2_Cl_2_ followed by HPLC-UV quantification. The assessed values are 6.18 wt % for SR610-PLLA, 7.48% for GM18-PLLA, and 6.31% for LT25-PLLA, which correspond to 1.6 mmol/g for SR610-PLLA, 2.9 mmol/g for GM18-PLLA, and 2.0 mmol/g for LT25-PLLA, respectively. The obtained values for the loaded compounds are in accordance with the nominal amount (10 wt % with respect to the polymer) and with those estimated by TGA measurements of the functionalized mats (Figure S3). In fact, PLLA and the β-lactams compounds exhibit different thermal stabilities: the polymer degraded in a single step between 200 and 350 °C, while β-lactams alone were characterized by a less defined (almost doubled) thermal degradation peak, with an onset at around 125 °C and residues at 600 °C on the order of 10–20% weight.

β-Lactam-PLLA samples displayed a two-step degradation, the first of low entity, which was attributed to the degradation of the compounds, followed by a more intense peak assigned to PLLA degradation. Since the degradation intervals of the plain components were partially superimposable, only an estimated loading of the β-lactams on PLLA could be calculated by TGA. The resulting values of about 8% weight for all three samples were substantially in agreement with the loading determined after dissolution by HPLC-UV analysis. Moreover, it is worth noting that the presence of β-lactams in GM18-PLLA and LT25-PLLA samples induced PLLA degradation at higher temperatures compared to the polymer alone, thus indicating a stabilization of the material, while this effect was not observed for the SR610-PLLA sample. HPLC-MS and ^1^H NMR analysis of the β-lactams after extraction from PLLA mats established the complete integrity of the compounds upon loading.

Calorimetric analyses were carried out on β-lactam-PLLA scaffolds and on plain PLLA for comparison (Figure S4). From the obtained DSC curves, the loading of the molecules did not appear to affect the thermal properties of PLLA, which resulted in a completely amorphous orientation after the electrospinning process, as previously reported.^24^ Indeed, PLLA is a slow crystallizable polymer, and given the high rate of fiber solidification during the electrospinning process, polymer chains have little time to organize in a crystal structure; therefore, PLLA crystallization is inhibited. Considering GM18-PLLA and LT25-PLLA samples, the presence of the β-lactam caused a plasticizing effect leading to a lowering of the glass transition temperature (*T*_g_ = 49 and 53 °C, respectively) compared to plain PLLA nanofiber (56 °C). Also, a slight decrease of Δ*H*_c_ and melting temperature (*T*_m_) was detected, indicating, respectively, a lower crystallization capability and the presence of a less perfect crystalline phase upon loading of β-lactams. This evidence might indicate that molecular interactions are present between PLLA and the two molecules, higher for GM18 than LT25, on the basis of the calorimetric data. The SR610-PLLA scaffold, on the other hand, showed a *T*_g_ similar to that of PLLA alone, indicating no plasticizing effect of the molecule, while the cold crystallization occurred at lower temperature than PLLA (110 °C vs 125 °C) and was characterized by a sharper peak, indicating a faster crystallization kinetics in SR610-PLLA compared to PLLA alone and to the other two functionalized scaffolds.

### Release Study in Aqueous Media

**Figure 3:**
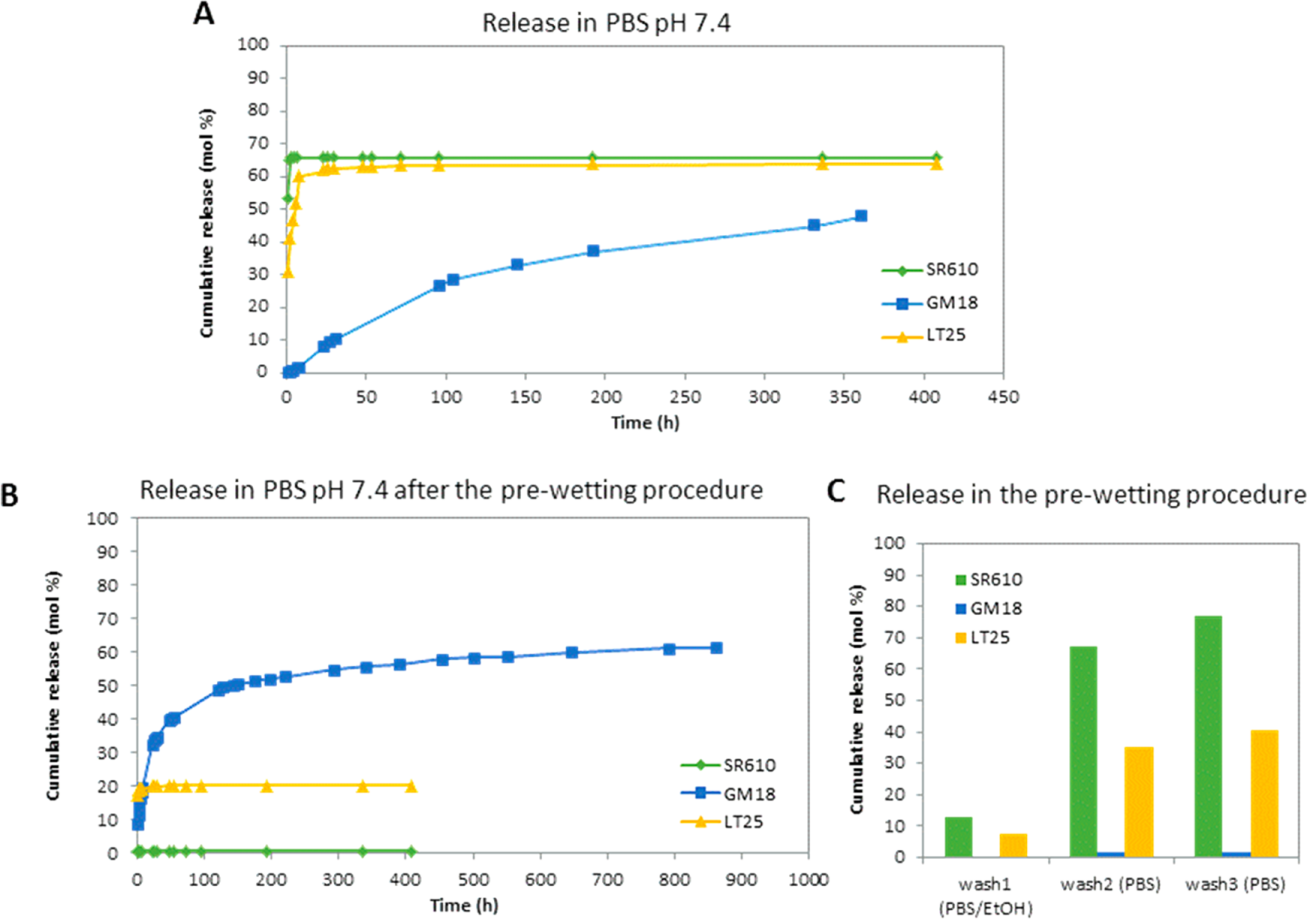
Release of β-lactams SR610 (green), GM18 (blue), and LT25 (yellow) from SR610-PLLA (6.18 wt %), GM18-PLLA (7.48 wt %), and LT25-PLLA (6.31 wt %) in phosphate buffer solution (pH 7.4). The scaffolds were used as such for the release studies (A) or with a prewetting treatment (B). The cumulative release is reported as mol % of the total loaded amount of β-lactams over time. The cumulative release during the wetting phase is also reported (C). Data were obtained in triplicate. Bars represent the mean values ± SD (standard deviation of the means) of results from three experiments.

The in vitro release of β-lactams SR610, GM18, and LT25 from the corresponding functionalized scaffolds SR610-PLLA (6.18 wt %), GM18-PLLA (7.48 wt %), and LT25-PLLA (6.31 wt %) was evaluated in phosphate buffer solution (PBS) at pH = 7.4 as a model for physiological conditions (Figure 3). The release data in triplicate were obtained by HPLC analysis of each refresh and expressed as cumulative release in mol %. Compound SR610—soluble in water (clogP = −0.24, calculated with specific algorithms from fragment-based methods developed by the Medicinal Chemistry Project of CambridgeSoft and BioByte in the ChemBioOffice suite)—showed a 50 mol % release in the first refresh, reaching a 64 mol % in the second refresh and less than 1 mol % in further refreshes. Compound LT25 (clogP = 0.92) was released in a 30 mol % amount in the first refresh and in a total 60 mol % within five refreshes. The release of SR610 and LT25 was monitored for additional 10 refreshes (400 h, 16.7 d), reaching a total released amount of around 64 mol % of the corresponding loaded β-lactams. Compound GM18 (clogP = 1.26) had a slower release in the initial four refreshes, probably due to its higher lipophilic character, indicated by the clogP that gives rise to a slower diffusion in the aqueous solution than SR610 or LT25.

This result is in agreement with calorimetric results reported above that were interpreted on the basis of a greater interaction between GM18 and PLLA. After the fifth refresh, compound GM18 exhibited a slow, sustained release reaching 48 mol % within 15 refreshes. From the collected data, we could estimate a release of 11 μg per refresh on average over the last six refreshes.

Notably, for all three scaffolds SR610-PLLA, GM18-PLLA, and LT25-PLLA, a complete release of the compounds from the PLLA scaffold was never observed, since from 25 to 50 mol % of the loaded β-lactams were held on the nanofibers after 15 days (360 h). The initial burst of SR610 and LT25 could be related to that portion of molecules at the PLLA fiber surface in direct contact with the aqueous medium, whereas those molecules contained within the inner part of the fibers, with less exposure to the aqueous medium, interact more strongly with PLLA and are progressively released during the steady state.

It is well-known among researchers working with electrospun scaffolds for tissue engineering applications that scaffolds need to be wetted in order to allow cells to access the pores.^24,25^ In order to test the effect of a prewetting procedure on the release of the β-lactams, a set of experiments were conducted after a prewetting treatment of SR610-PLLA, GM18-PLLA, and LT25-PLLA samples in aqueous ethanol solutions to improve the swelling of the material. The release experiments were then conducted as described above, and the amounts of the released β-lactams were evaluated by HPLC-UV. SR610-PLLA released 80 mol % of the β-lactam during the prewetting treatment, possibly due to its high hydrophilicity (Figure 3C, wash 1–3); accordingly, when subjected to the release in PBS pH 7.4, only traces of the molecule were detected in the next 6 refreshes, for a total of 17 days. LT25-PLLA sample showed a similar effect with a 40 mol % release of LT25 in the prewetting treatment; an additional 20% was delivered during the release in PBS pH = 7.4 (17% in the first refresh and around 3% in the next 14 refreshes for a total of 17 days). Despite the substantial released amounts of SR610 and LT25 during the pretreatment, it is worth mentioning that a complete release of the compounds from the PLLA scaffolds was not observed also in this case. Indeed, a residual 20 mol % of SR610 and 40 mol % of LT25 are still available on the mats for subsequent steady releases.

Conversely, the prewetting treatment did not influence the release of GM18, since only traces of the compound were detected in the pretreatment and its release profile was mainly in accordance with that detected without prewetting; in fact, after 15 refreshes, the released amount of GM18 was around 50 mol % of the initial content. The release from GM18-PLLA mat was monitored for a longer time, providing a constant profile on releasing 1.6 μg of GM18 per refresh (average refreshes 15–30) and 60 mol % of the total loaded compound after 35 days. In this case, a low delivery of GM18 could be favorable for maintaining an active and constant concentration of the molecule in the physiological environment, thus enabling a longer activity of the biomaterial.

The release of compound GM18 from GM18-PLLA mats was additionally studied in Milli-Q-H_2_O and acetate buffer 0.1 M at pH = 5. A comparison of GM18 release among the three different aqueous media (PBS pH = 7.4, Milli-Q-H_2_O, acetate buffer pH = 5) is reported in Figure S5. The release of GM18 in acetate buffer showed a slow initial release similar to that in PBS pH = 7.4, and after 15 refreshes, a total amount of 74 mol % of GM18 was recovered. The release in Milli-Q-H_2_O turned out to be faster compared to the buffered solutions, with a total recovery of 45 mol % in seven refreshes, followed by a slower trend with about 60% of released compound after 37 days.

The scaffolds SR610-PLLA, GM18-PLLA, and LT25-PLLA after the release experiments in PBS were subject to SEM analysis, and in all samples no modification of the fiber morphology was detected. The images confirmed the preservation of the fibrous matrix, which remained characterized by good homogeneity and absence of defects (Figure S6).

### Biological Characterization of Functionalized PLLA Scaffolds

Using the functionalized PLLA scaffolds described above, the cell response was investigated at different levels, including attachment, morphology, translation of proteins typically involved in cell adhesion process, and gene expression of integrin subunits in cell proliferation. hBM-MSCs, which are known to express high levels of different integrin subunits,^26^ including the targets of β-lactam agonists proposed here, were selected for this purpose.

### Cell Adhesion and Morphology Assessment onto Functionalized PLLA Scaffolds

**Figure 4:**
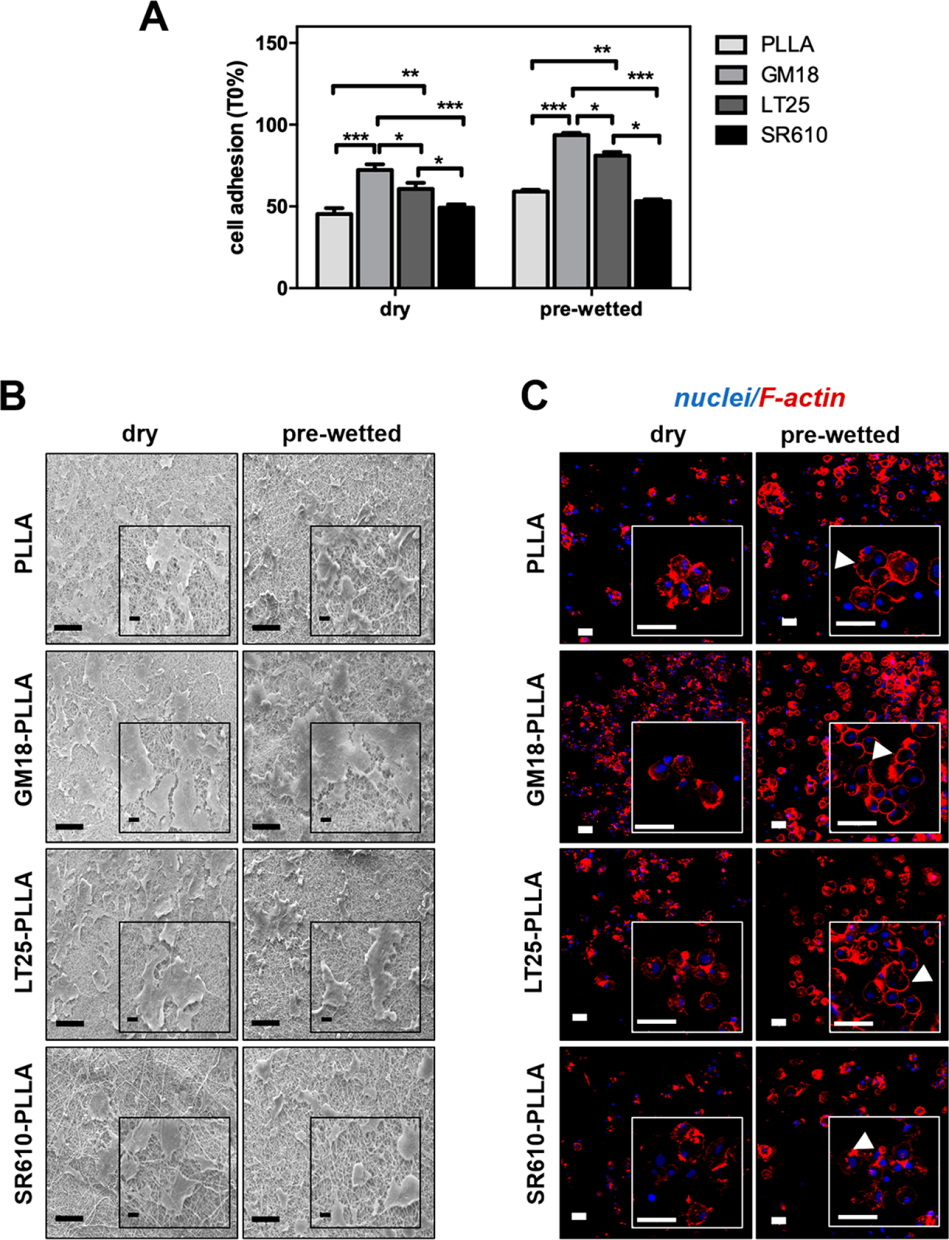
hBM-MSC attachment onto plain PLLA and β-lactam-PLLA scaffolds SR610-PLLA (6.18 wt %), GM18-PLLA (7.48 wt %), and LT25-PLLA (6.31 wt %). (A) Cell adhesion was analyzed after 2 h from seeding and was plotted as a percentage of viable cells in comparison with initial seeded cell number (*T*_0_). Bars represent the mean values ± SD (standard deviation of the means) of results from three experiments (*n* = 3, *** *p* < 0.001; ** *p* < 0.01; * *p* < 0.05). (B) Cell morphology assessed by SEM on all samples. Scale bars = 100 μm and magnification 500×. Inset scale bar = 10 μm and magnification 1.5×. (C) Cell morphology assessed by CLSM as described in the Experimental Section. The cytoskeleton organization was observed by F-actin staining with phalloidin (red). Nuclei were stained with Hoechst 33342 (blue). Magnified areas of cells are shown in insets. Arrows indicate F-actin distribution. Scale bars: 50 μm. All data shown in (B) and (C) are representative images from 3 independent experiments.

The attachment, adhesion, and spreading are typical processes of the first phase of cell/material interactions. Many reports proved that the immobilization into the scaffold of a defined spectrum, concentration, spatial distribution, and controlled release of bioactive molecules, such ligands against the receptors on cell surface, proteins, growth factors, hormones, and enzymes or synthetic regulators of cell behavior, could be a winning strategy to modulate scaffold bioactivity and its interaction with the cell, thereby promoting their adhesion, growth, and differentiation inside the material.^27^ As a starting point, hBM-MSC adhesion was verified in agonist-functionalized scaffolds in both dry and prewetting conditions after 2 h of incubation (Figure 4A and Figure S7A) in order to allow sufficient adhesion of the cells to the scaffolds,^28^ but with the lowest possible compound release (see Figure 3).

Among β-lactams, the most potent in enhancing cell attachment turned out to be GM18 (Figure S8 and S9, respectively), which was also associated with a very low release from PLLA fibers (see Figure 3). Consistently, in both cases (dry and prewetted), a higher cell adhesion was measured in GM18- and LT25-PLLA in comparison with the plain PLLA surface (*** *p* < 0.001 and ** *p* < 0.01 vs PLLA, respectively) (Figure 4A). Moreover, a significant enhancement of cell adhesion was observed in GM18-PLLA than LT25- and SR610-PLLA scaffolds (* *p* < 0.01 and *** *p* < 0.001, respectively), and a significant difference between LT25- and SR610 (* *p* < 0.05) was detected (Figure 4A). Not surprisingly, due to the highest SR610 release from PLLA fibers, no substantial change in the cell adhesion was assessed in SR610-PLLA scaffolds as compared with plain PLLA (*p* > 0.05) (Figure 4A).

Remarkably, the prewetting treatment led to a significant increase of cell adhesion as compared to the dry one, except for the SR610 sample (*p* > 0.05) (Figure S7A), consistent with literature findings showing an enhanced cell spreading on hydrophilic surfaces compared to hydrophobic surfaces by using different cell types.^29^

Coherently with agonist-release activity and viability data, both SEM and confocal laser scanning microscopy (CLSM) displayed that GM18-PLLA appeared to be the most cell-populated scaffold in both dry and prewetting treatments (Figure 4B and C), whereas LT25- and SR610-PLLA showed almost the same cell density and morphology of plain PLLA.

SEM images revealed a robust spreading of flattened-shape cells covering GM18-PLLA, differently from the randomly dispersed cells onto the other PLLA scaffolds (Figure 4B). In synergy with other scaffolds cues (e.g., chemistry modifications), substratum surface topography could deeply influence cell spreading over time and polarization, affecting the focal adhesion formation, cytoskeleton organization (e.g., F-actin distribution), and cell morphology acquisition.^30^ Accordingly, as clearly evidenced by F-actin staining after 2 h of incubation, cells grew onto TCPS (Figure S7C), appearing with spindle-shaped morphology showing abundant and organized long actin stress fibers. A different scenario was observed at the same time of culture in all agonists functionalized- and plain- PLLA scaffolds, on both dry and prewetting condition (Figure 4C). Indeed, in the former condition, cells displayed a rounder shape, and adhesion seemed to be at an early stage with a disordered F-actin staining throughout the cell body, which is clear evidence of a less organized F-filament network (Figure 4C and Figure S7C). In prewetted conditions, seeded cells in all analyzed PLLA showed rounded protrusions, exhibiting pronounced F-actin fiber concentration mostly confined toward the cell edges (Figure 4C, arrows) that proved the cells attempt to form contacts with fibrous architecture of the scaffolds. However, in both conditions, no marked differences in cell morphology were observed among plain- and functionalized-PLLA scaffolds, although the surface modification using agonists was an effective way to enhance hBM-MSCs adhesion on PLLA (Figure 4A). Based on these findings, most likely the physical architecture nanofibers creating a filament weft was most effective than the surface chemistry, able then to elicit some effects on the actin cytoskeleton that could be decisive for cellular spreading, morphology phenotype, and fate.^31^

### Evaluation of Proteins Involved in the Cell Adhesive Process onto Functionalized PLLA Scaffolds

**Figure 5:**
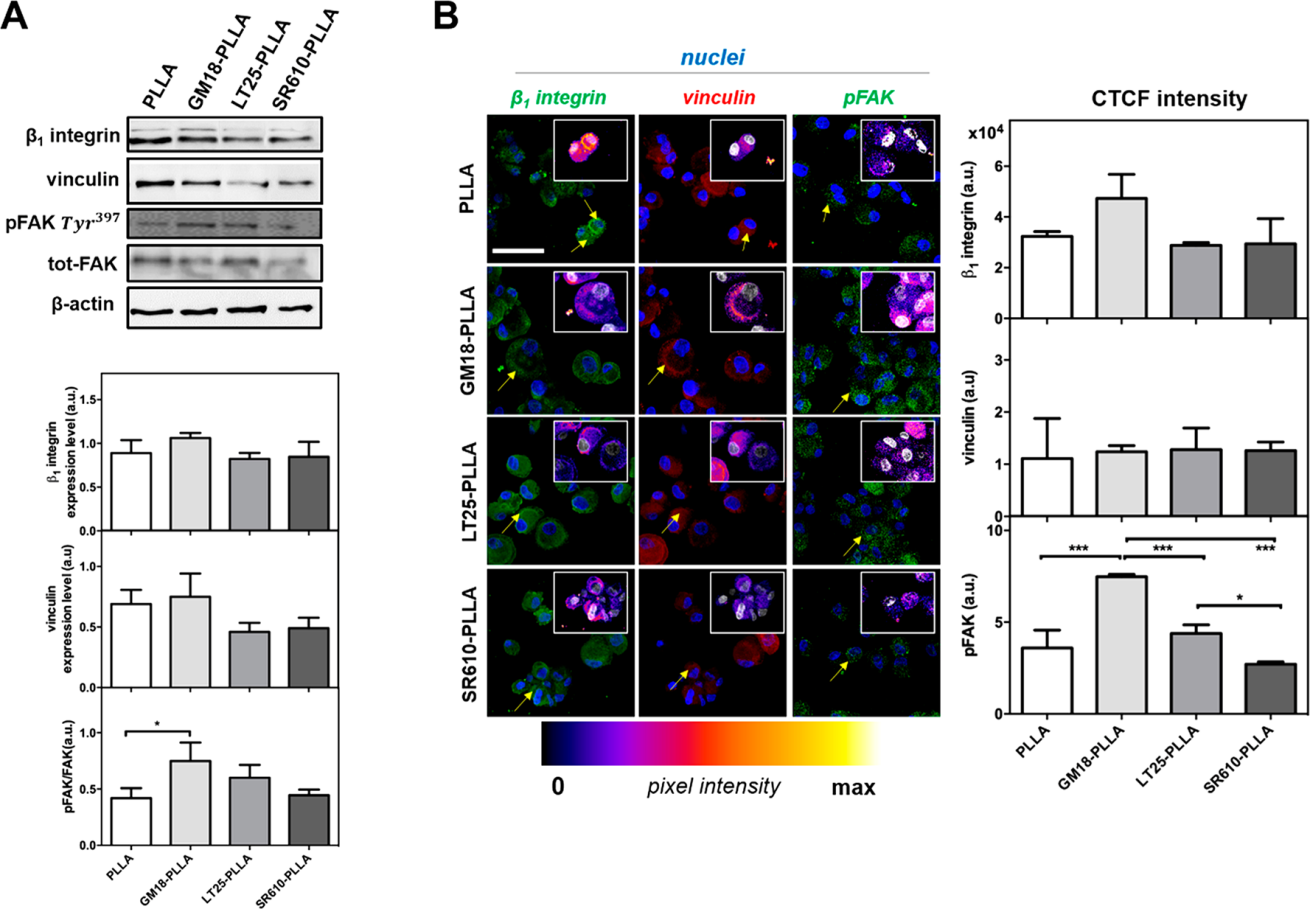
Qualitative and quantitative analysis of specific proteins involved in the cell adhesive process to plain PLLA and SR610-PLLA (6.18 wt %), GM18-PLLA (7.48 wt %), and LT25-PLLA (6.31 wt %) after 2 h from seeding. (A) Western blotting analysis. Bar graphs show β_1_ integrin and vinculin expression level obtained normalizing to the β-actin housekeeping protein signal. The activation level of FAK was presented as a ratio between the phosphorylated and total FAK protein after normalization to β-actin. Bars represent the mean values ± SD of results from three experiments (*n* = 3). Statistical significance values are indicated as *** *p* < 0.001 and * *p* < 0.05. (B) CLSM images, showing the expression of focal adhesion β_1_ integrin (green, 488 Alexa Fluor), vinculin (red, 633 Alexa Fluor), and p-FAK (green, 488 Alexa Fluor) on different PLLA scaffolds, were acquired at 40× magnification. Nuclei were stained with Hoechst 33342 (blue). Scale bars: 50 μm. Yellow arrows indicated protein distribution at cellular level. The insets display a protein staining with false coloring from dark purple to bright yellow by use of the fire lookup table (LUT) scheme to highlight differences in the intensities of the signals obtained with ImageJ software. Graphs show the correct total cell fluorescence intensity (CTCF) measured in each sample (*n* = 3, *** *p* < 0.001 and * *p* < 0.05).

Cells can mainly sense mechanical cues from the substrates via integrins receptors.^32^ After ligand binding, the entire process is very closely related to the rapid formation of the so-called “focal adhesions” (FAs) sites. At these distinct sites, integrins are recruited and communicate with scaffolding proteins (e.g., α-actinin, talin, paxillin, and vinculin) and signaling kinases (e.g., integrin-linked kinase, focal adhesion kinase (FAK)), that binding directly or indirectly through actin-binding proteins to the cytoplasmic domains of integrins, constitute a bridge between integrins and cytoskeleton, which produces a signal transduction inside the cells able to influence cell behavior and fate.^32^ The immobilization of integrin-specific ligands to substrates is usually used to promote integrin-dependent cell adhesion, including the recruitment of structural proteins and the activation of signaling molecules.^27d,33^ Similarly, surface chemistry modulates focal adhesion assembly and focal adhesion kinase phosphorylation.^22,34^ Hence, to assess the activity of the integrin-agonist functionalized PLLA, specific molecules involved in the interaction process were analyzed. Since wettability was found to be quite important for cell adhesion on agonist-PLLA and plain PLLA scaffolds, studies were conducted exclusively in prewetting conditions by quantitative and qualitative analyses after 2 h of incubation (Figure 5A and B, respectively). Similarly, the same experiments were performed in hBM-MSCs cultured in TCPS control (Figure S10). β_1_ integrin is a ubiquitously expressed subunit with an important role in the formation of focal contacts and interaction with cytoskeleton.^26a,35^ Vinculin is a component of focal adhesions and adheren junctions, which responds to and transmits force at integrin- and cadherin-containing adhesion complexes to the cytoskeleton.^36^ Both proteins are also recruited during the interaction of cells, including stem cells, with materials.^37^ In this study, as revealed by quantitative immunoblotting data (Figure 5A), no noticeable differences in the expression of β_1_ integrin and vinculin were determined by incubating for 2 h hBM-MSCs on the different scaffolds (*p* > 0.05). The focal adhesion kinase FAK is a key downstream component in integrin-mediated signaling. FAK, interacting through the C-terminal region (containing the FAT (focal adhesion targeting) domain with proteins of the focal adhesion complex and through the N-terminal domain with the β_1_ subunit of integrins, it is involved in the integrin-mediated responses, such as cellular motility, adhesion, proliferation, and protection against apoptosis and differentiation.^38^ The phosphorylation of FAK was then assessed on all samples (Figure 5A). On PLLA, LT25-PLLA, and SR610-PLLA, the pFAK signals were at low levels (Figure 5A), while pFAK expression on GM18-PLLA was more upregulated than the other groups (* *p* < 0.05).

This behavior matched the release profile of β-lactams from the fibers well (Figure 3), further emphasizing the active role of the agonist GM18 in making suitable PLLA scaffolds for an early cell/biomaterial interaction. Integrin engagement and clustering are early steps in the formation of cell–substrate adhesions, which are accompanied by the recruitment and activation of the various components of the mechano-sensing system of integrin-based FA, such as vinculin and FAK proteins, at the level of cellular plasma membrane.^39^ Then, the distribution of downstream β_1_ integrin, vinculin, and FAK was tested on all samples by immunofluorescence investigation.

In accordance with the above quantitative results, immunofluorescence results (Figure 5B) did not show significant difference in the fluorescence signal intensity of β_1_ integrin and vinculin among the scaffolds, while significant changes in the fluorescence distribution inside the cells were appreciable for both proteins. β_1_ integrin signal appeared more marked at the membrane level of cells seeded on GM18-PLLA and LT25-PLLA than those on plain- and SR610-PLLA, in which, by contrast, the fluorescence was widespread mainly at the cytoplasm level (Figure 5B, see yellow arrows). A similar trend was also found for vinculin that displayed a detectable signal mostly in proximity of the cell membrane on GM18-PLLA (Figure 5B, see yellow arrows), suggesting a larger extent of focal adhesion formation and maturation. Strikingly, on GM18-PLLA samples, the sites of vinculin enrichment were indeed correlated with peripherical regions in the cell showing more β_1_ integrin, still proving a higher activation of integrin-dependent adhesion in comparison with the other scaffolds. Consistently, although the presence of the typical “spots” of pFAK staining was found distributed throughout, at both the central and peripheral regions on plain- and functionalized-PLLA scaffolds (Figure 5B), it was possible to detect a significant enhancement of activated-FAK fluorescence mainly on the cells adhered on GM18-PLLA scaffolds in comparison with those cultured on the other groups (*** *p* < 0.001 vs PLLA, LT25- and SR610-). As expected, no significant changes were obtained between PLLA alone, LT25-PLLA, and SR610-PLLA scaffolds (*p* < 0.05); on the contrary, a significant difference was measured between LT25-PLLA and SR610-PLLA scaffolds, still in agreement with release studies (Figure 5B). Taken together and in line with literature,^40^ these findings suggest that changes in focal adhesion protein distribution and FAK increased activation observed in the cells adherent onto functionalized-PLLA scaffolds were clearly related to the β-lactam incorporation, which may be responsible for a higher adhesion and spreading of hBM-MSCs.

### Cell Proliferation Assessment onto GM18-Functionalized PLLA Scaffold

**Figure 6:**
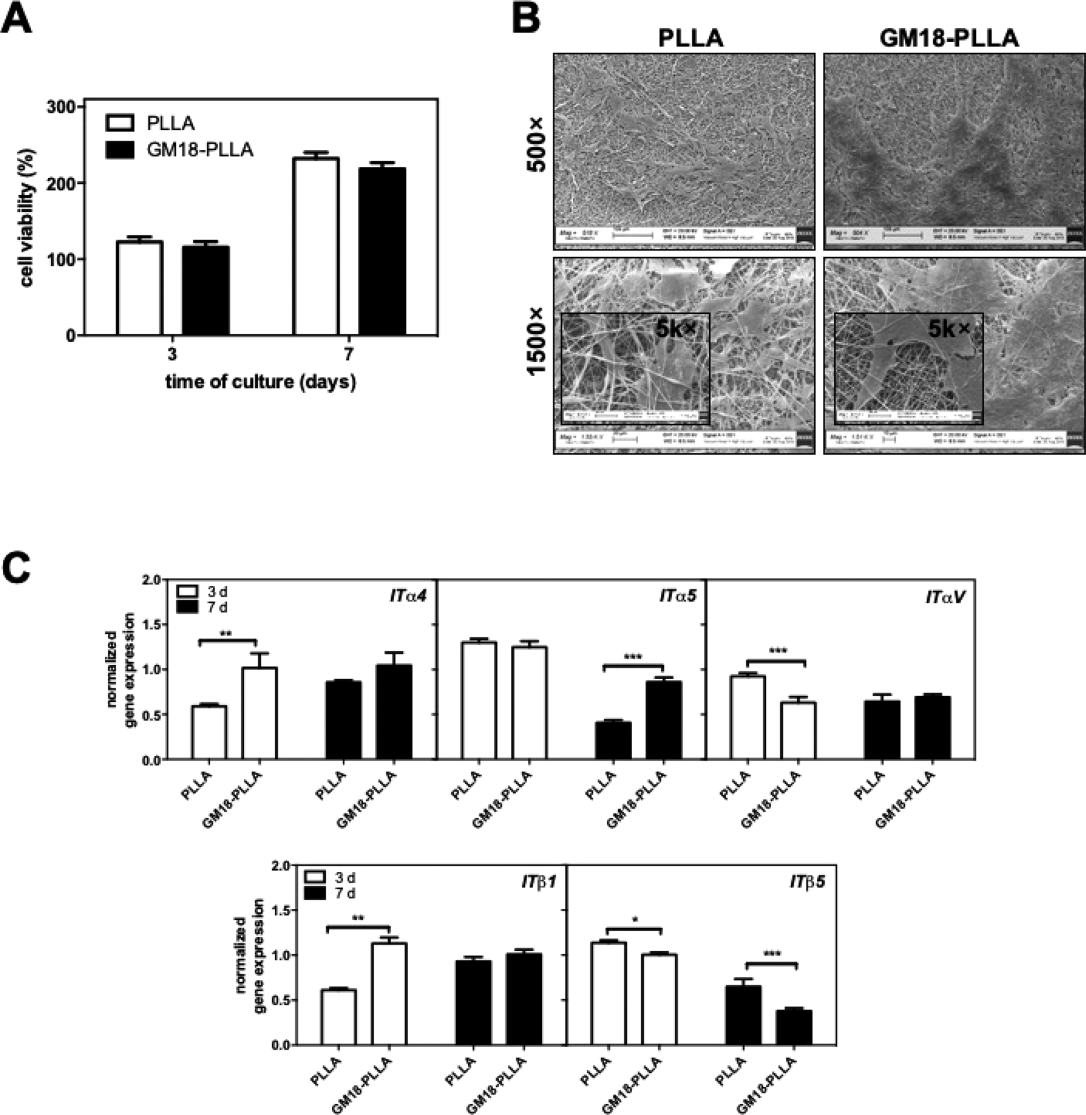
hBM-MSCs viability and morphology on PLLA and GM18-PLLA (7.48 wt %) scaffolds after prewetting treatment. (A) Cell viability was evaluated at day 3 and 7, respectively. Cell viability was plotted as the percentage of viable cells in comparison with initial state (day 0 = *T*_0_) set as 100% cell viability. Bars indicate mean values ± SD of the mean of results from three experiments. (B) Representative SEM images of hBM-MSCs cultured on PLLA and GM18-PLLA at day 7 of culture. (C) Gene expression of the indicated integrin subunits. The graphs show the fold increase of gene expression related to cells during the initial state (day 0), set equal to 1 (*n* = 3). Statistical significance values are indicated as ** *p* < 0.01; *** *p* < 0.001.

To date, various strategies have been developed to improved knowledge of integrin biology and performance of biomedical devices for tissue engineering purpose.^41^ it well-known that once a cell attached firmly to the material through integrin coupling, integrin signaling converges with growth factor signaling in the activation of ERK signaling cascade, which finally impacts proliferation by the activation of cyclin D1, a key regulator the G1–S cell cycle transition.^42^ hBM-MSCs growth was assessed on all functionalized PLLA and plain PLLA. However, significant differences were observed uniquely between PLLA and GM18: the substrate also displayed improved cell adhesion in comparison with plain-PLLA. By contrast, LT25 and SR68 did not show significant differences in cell proliferation over plain PLLA, in agreement with release and cell adhesion studies (data not shown). Interestingly PLLA and GM18-PLLA scaffolds showed a comparable viability at both 3 and 7 days of cell culture (Figure 6A).

However, the determination of the cell proliferation rate, defined as the increase in the ratio of cell number at days 1 and 7 over the adherent cells number, revealed some differences: it was greater on PLLA in comparison with GM18-PLLA (∼2.0 vs 1.4 at day 3 and ∼3.9 vs 2.4 at day 7), indicating a lower cell proliferating stage in the latter that may be a consequence of a higher cell adhesion due to GM18 incorporation. Indeed, despite equal cell growth area, a greater adherent cell number was determined in GM18-PLLA than PLLA at 2 h which, over the next few days, may lead growing cells to become quickly confluent, eliciting in these cells an early ending or reducing cell division event compared to those on plain PLLA. SEM images seem to support this statement, displaying a widespread cell monolayer over the GM18-PLLA scaffold and a minor number of adherent cells with flattened shape homogeneously covering the PLLA scaffold (Figure 6B).

Integrins regulate, in a cooperative manner, cell cycle and the expression of gene related to differentiation. Moreover, external cues can trigger cell adhesion, migration, proliferation, and differentiation by changing the expression and activation of specific types of integrin subunits and heterodimers as well as the engagement of cytoskeletal protein.^43^ Studies on different cell systems, including human mesenchymal stem cells, have also found that the engagement of the integrin receptor repertoire differs according to surface characteristics, thereby influencing deeply the cell behavior and phenotype.^44^ Nevertheless, contradictory results exist, and it is still unclear the role of the specific integrin subunits in hMSCs proliferation and differentiation after the interaction with the surfaces.^45^ Thus, the β_1_ subfamily of integrins seem to be the most important subunits for hMSC–material interaction. Indeed, by binding many α-subunits, such as α_1_, α_2_, α_3_, α_4_, α_5_, α_6_, α_V_, and α_9_, β_1_ modulates the spreading, the adhesion strength, the proliferation, and the differentiation of human bone marrow cells on fibronectin, collagen, laminin, and different types of biomaterials.^46^ Similarly, integrin α_V_, assembling with subunits β_1_, β_3_, β_5_, β_6_, or β_8_, is thought to play a critical role in the above processes.^47^ The association of α_v_β_3_ integrins (a classical vitronectin receptor) with adapter proteins downstream of growth factor receptors is thought to be required for sustaining growth factor activation of downstream proliferative signals and long-term mitogenic pathways.^48^ Furthermore, together with α_v_β_5_, known mainly to bind vitronectin and bone sialoprotein in ECM,^46d^ α_v_β_3_ participates in the strong cell-attractive responses and mitogenic ability of substrate-bound and soluble tropoelastin.^49^ Among the family of integrins, α_4_β_1_, which mediates cell–cell and cell–extracellular matrix interactions through adhesion to the vascular cell adhesion molecule (VCAM)-1 and to the IIICS region of fibronectin,^50^ has been reported to be implicated in the homing of not only the hematopoietic stem cells and metastatic tumor cells but also the hBM-MSCs cells.^51^ These findings led us to hypothesize an innovative therapeutic approach for tissue regeneration concerning the engagement of specific integrins present on the MSCs surface for moving the MSCs to the material surface. Likewise, the activation and abundance of specific integrins can alter and modulate the integrin composition of cell–matrix adhesions during development, angiogenesis, wound healing, and cancer progression,^52^ which is then an aspect to take into account for further interpretation of outcomes and clinical applications.

With this in mind, to better investigate the performance of GM18-PLLA scaffolds and their effects on cell behavior, the gene expression of α_4_ and β_1_ subunits (both targets of GM18 agonist) were analyzed by RT-qPCR at two culture times. The samples were also analyzed for the gene expression of three other additional integrin subunits, α_V_, α_5_, and β_5_ (Figure 6C). RT-qPCR results showed that the presence of GM18 agonist on PLLA leads to significant changes in integrin expression with different temporal patterns.

GM18 is a specific agonist for α_4_β_1_ integrin. Interestingly, at 3 days of culture, a significant increase in α_4_ expression was found in cells adherent on GM18-PLLA (Figure 6C), with a concurrent upregulation of β_1_ that probably ensured enough numbers of surface receptors to translate agonist stimulus into a cell response, still pointing out the effective action of GM18 as cell-attractive molecules. At day 7 of incubation, the absence of a substantial difference between GM18-PLLA and PLLA scaffolds in both α_4_ and β_1_ genes may be linked with reaching the cell confluent state in GM18 enriched PLLA fibers because of a great cell adhesion in the initial phases of interaction (Figure 6A and B), thus indirectly proving the positive effects of GM18 incorporation. Besides, the greater cell colonization of the GM18-PLLA scaffold might also be responsible for the downregulation of α_V_ and β_5_ expression; as stated above, both involved in hMSCs adhesion and homing (Figure 6C), which could be an indication of a “full adhesion state” reached by the cells onto this scaffold at the culture times considered. As well, the formation of compact cell monolayers over the GM18-PLLA surface may indeed inhibit cell growth and be responsible for α_5_ integrin mRNA expression. Integrin signaling is also critical in cellular differentiation.^53^ For example, the upregulation of α_5_ integrin is required for MSC osteogenic differentiation.^54^ Consistent with the higher cell adhesion and quickly cell confluence reached, at 3 days of culture, α_5_ integrin was upregulated on both plain PLLA and GM18-PLLA, but then downregulated at 7 days from the initial state (Figure 6C). Notably, at 7 days, the α_5_ subunit level was higher on GM18 than on plain-PLLA: this finding may be explained by a molecular connection between the adhesion/proliferation stimulated by GM18 and the activation of osteogenic differentiation genes. Certainly, the overlap of intracellular signaling cascades shared by agonist-PLLA triggered integrins and culture media activated-growth factors receptors might play a crucial role in the exchange of information throughout the human mesenchymal layer onto PLLA scaffolds that in turn could affect the expression and production of specific genes and proteins. Consequentially, additional experiments are needed to define the intracellular responses to GM18 incorporation onto PLLA scaffolds and clarify the biological action for its hMSCs modulation in term of cell adhesion, proliferation, and differentiation toward a specific biomaterial or tissue.

## Conclusions

In summary, we realized new functionalized biomaterials based on electrospun PLLA and monocyclic β-lactam compound agonist ligands of specific integrins. Incorporation into PLLA and release of the β-lactams were deeply investigated, and the new functionalized scaffolds were fully characterized. The new functional scaffolds were tested in enhancing adhesion of hBM-MSCs, focusing on their contribution in guide expression of specific adhesion proteins and proliferation. SEM, CLSM, and Western Blot analyses revealed that the presence of β-lactam agonists positively affected stem cell response to attachment onto PLLA scaffolds. Importantly, the β-lactam GM18 showed the best results, also supporting the enhanced cell proliferation onto PLLA over time. Incorporation of β-lactam into PLLA scaffolds can stimulate specific adhesion pathways, thus promoting the establishment of strong cell attachment that in turn elicits the cell proliferation activation. These findings suggest that β-lactam agonists could be added advantageously and safely to biomaterials for effectively improving stem cell colonization, endowed with interesting bioactive properties. The functionalized biomaterials might hold potential for tissue engineering applications, in the regenerative medicine field.

## Supporting Information Available

Scheme for the synthesis of β-lactams (Figure S1); fiber diameter of the investigated samples (Table S1); ATR-FTIR spectra (Figure S2a-c); thermogravimetric curves of the functionalized PLLA fibers (Figure S3); DSC analyses (Figure S4); thermal properties (Table S2); release of β-lactam GM18 in aqueous solution (Figure S5); SEM images of functionalized samples (Figure S6); cell adhesion of hBM-MSCs cultured on plain PLLA and agonist-PLLAscaffolds (Figure S7); evaluation of cell adhesion mediated by β-lactams GM18, LT25, and SR610 agonists on hBM-MSCs in the presence of fibronection (Figure S8) or in the absence of fibronectin (Figure S9); qualitative and quantitative analysis of integrin, vinculin and pFAK (Figure S10); primer sequences used for real-time qPCR (Table S3) (PDF)

## Abbreviations

CCR2: CC chemokine receptor 2
CCL2: CC chemokine ligand 2
CCR5: CC chemokine receptor 5
TLC: thin layer chromatography
