# Supplementary material for: Combining Biologically Active β-Lactams Integrin Agonists with Poly(l-lactic acid) Nanofibers: Enhancement of Human Mesenchymal Stem Cell Adhesion
Source: Biomacromolecules. 2020 Feb 3;21(3):1157–70. doi: 10.1021/acs.biomac.9b01550 (PMC7997109; doi:10.1021/acs.biomac.9b01550)
Supplement: Supplementary file 1 — bm9b01550_si_001.pdf [file bm9b01550_si_001.pdf]

Supporting Information

Combining Biologically Active β-Lactams Integrin Agonists with Poly(L-lactic acid) Nanofibers: Enhancement of Human Mesenchymal Stem Cell Adhesion

Giulia Martelli, Nora Bloise, Andrea Merlettini, Giovanna Bruni, Livia Visai, Maria Letizia Focarete* and Daria Giacomini*

Content

- Scheme for the synthesis of β-lactams (Figure S1) page 2
- Fiber diameter of the investigated samples (Table S1) page 2
- ATR-FTIR spectra (Figure S2a-c) pages 3-5
- thermogravimetric curves of the functionalized PLLA fibers (Figure S3) page 6
- DSC analyses (Figure S4) pages 7-8
- thermal properties (Table S2) page 8
- release of β-lactam GM18 in aqueous solution (Figure S5) page 9
- SEM images of functionalized samples (Figure S6) page 10
- cell adhesion of hBM-MSCs cultured on plain PLLA and agonist-PLLA scaffolds (Figure S7) page 11
- evaluation of cell adhesion mediated by β-lactams GM18, LT25, and SR610 agonists on hBM-MSCs in the presence of fibronection (Figure S8) or in the absence of fibronectin (Figure S9) pages 12-14
- qualitative and quantitative analysis of integrin, vinculin and pFAK (FigureS10) pages 14-15
- primer sequences used for real-time qPCR (Table S3) page 15

Scheme for the synthesis of β-lactams.

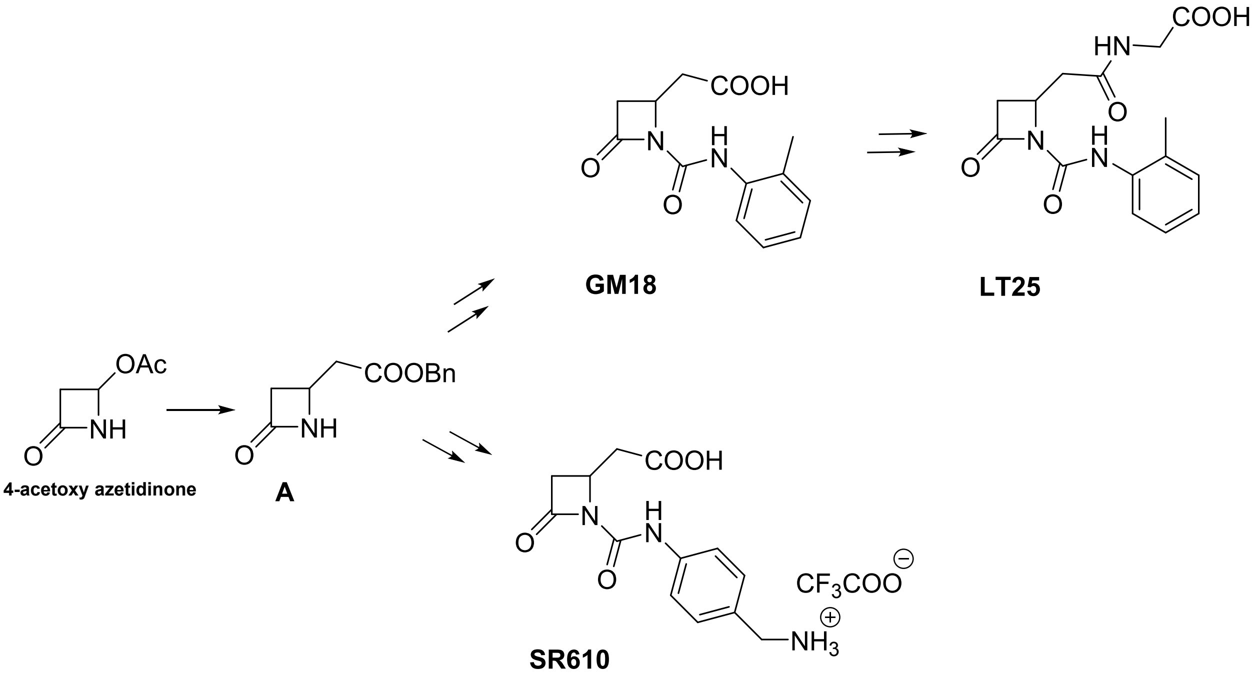

Figure S1. Synthetic paths for the synthesis of the three β-lactam compounds selected for this study. The procedures are described in ref. 8

Table S1: Fiber average diameter of the investigated samples

Sample	Mean diameter ± standard deviation (μm)
PLLA	0.6 ± 0.2
SR610-PLLA	0.44 ± 0.08
GM18-PLLA	0.5 ± 0.1
LT25-PLLA	0.5 ± 0.1

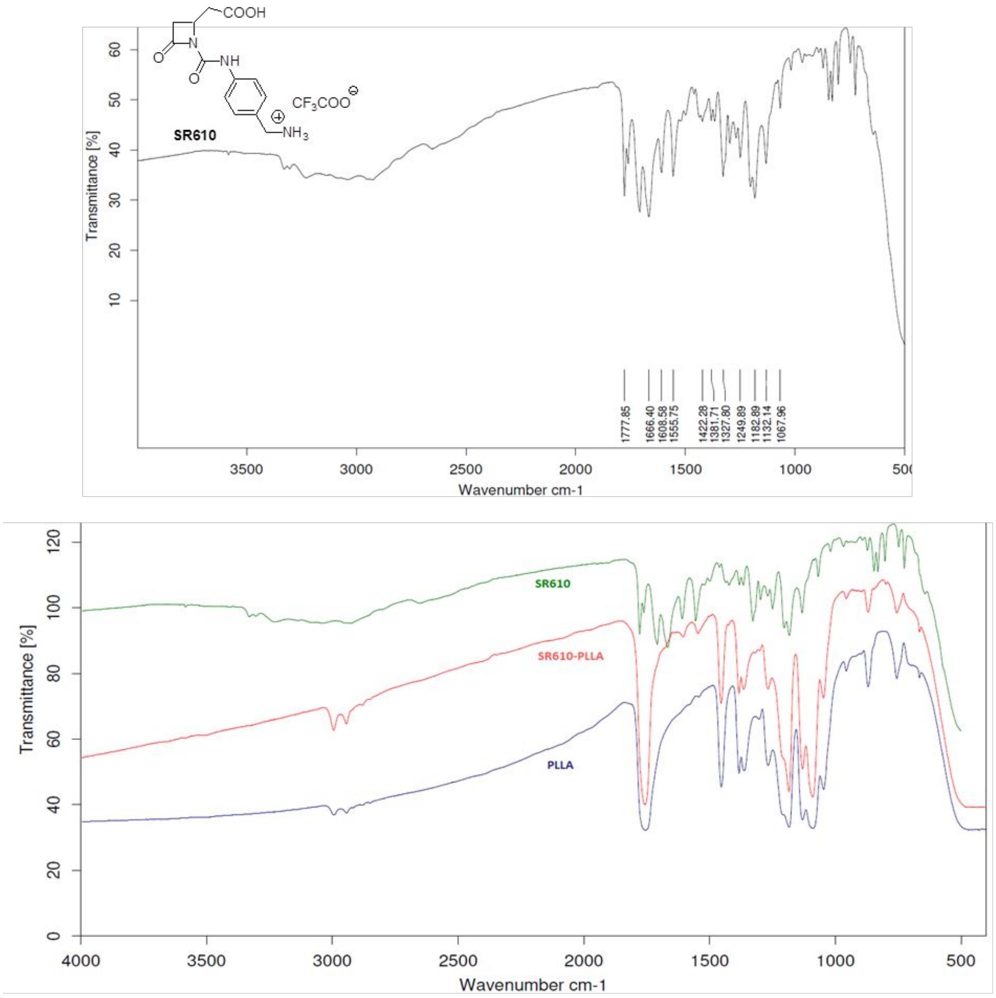

Figure S2a. ATR-FTIR spectra of compound SR610 and overlapped spectra of SR610, SR610-PLLA and PLLA alone.

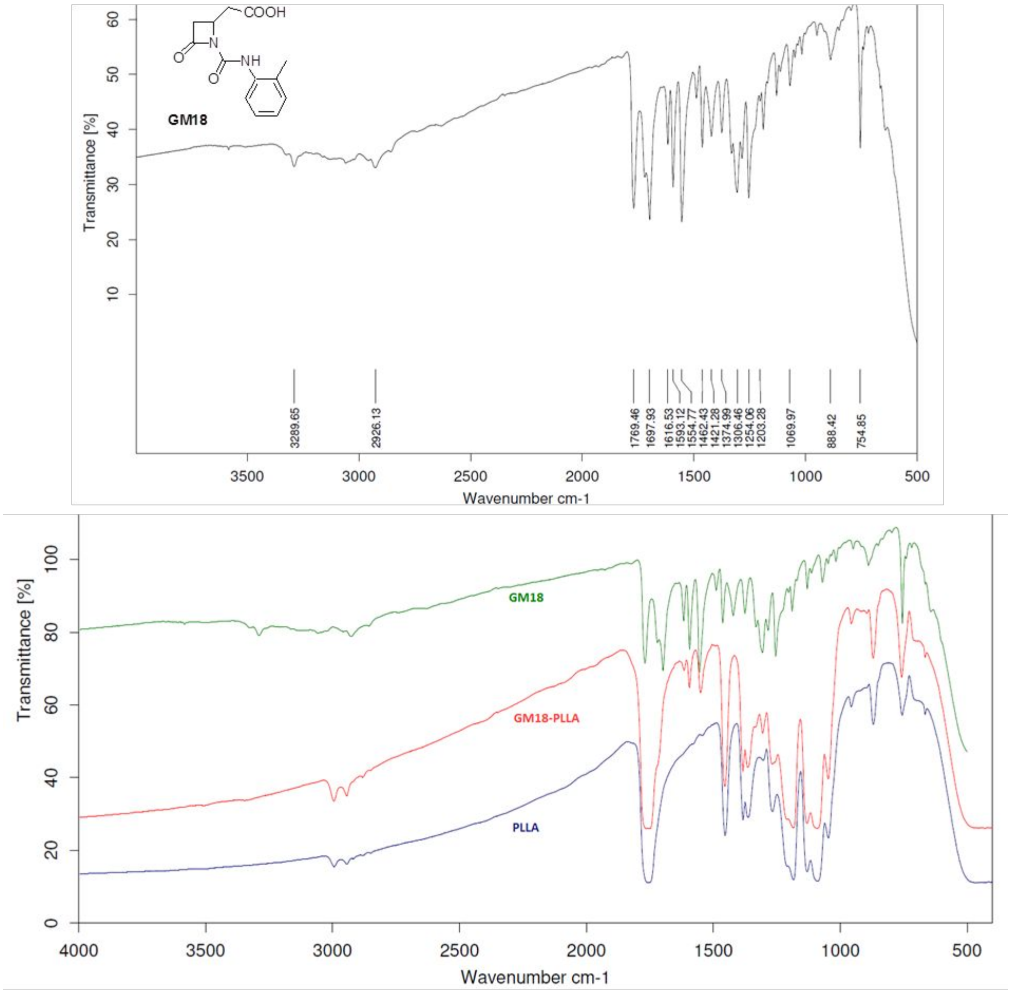

Figure S2b. ATR-FTIR spectra of compound GM18 and overlapped spectra of GM18, GM18-PLLA and plain PLLA

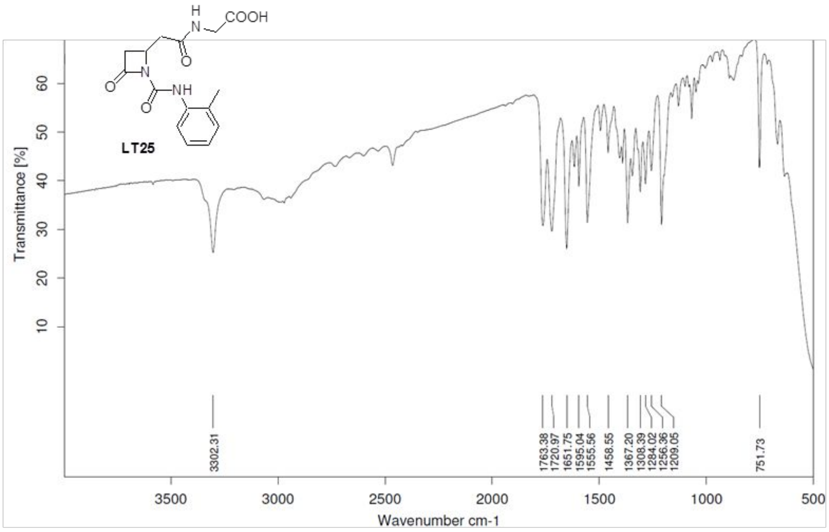

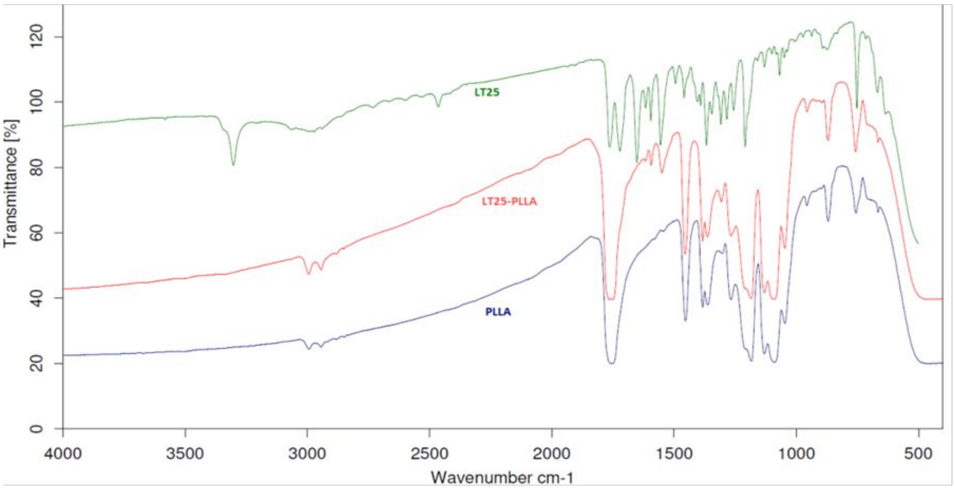

Figure S2c. ATR-FTIR spectra of compound LT25 and overlapped spectra of LT25, LT25-PLLA and PLLA alone

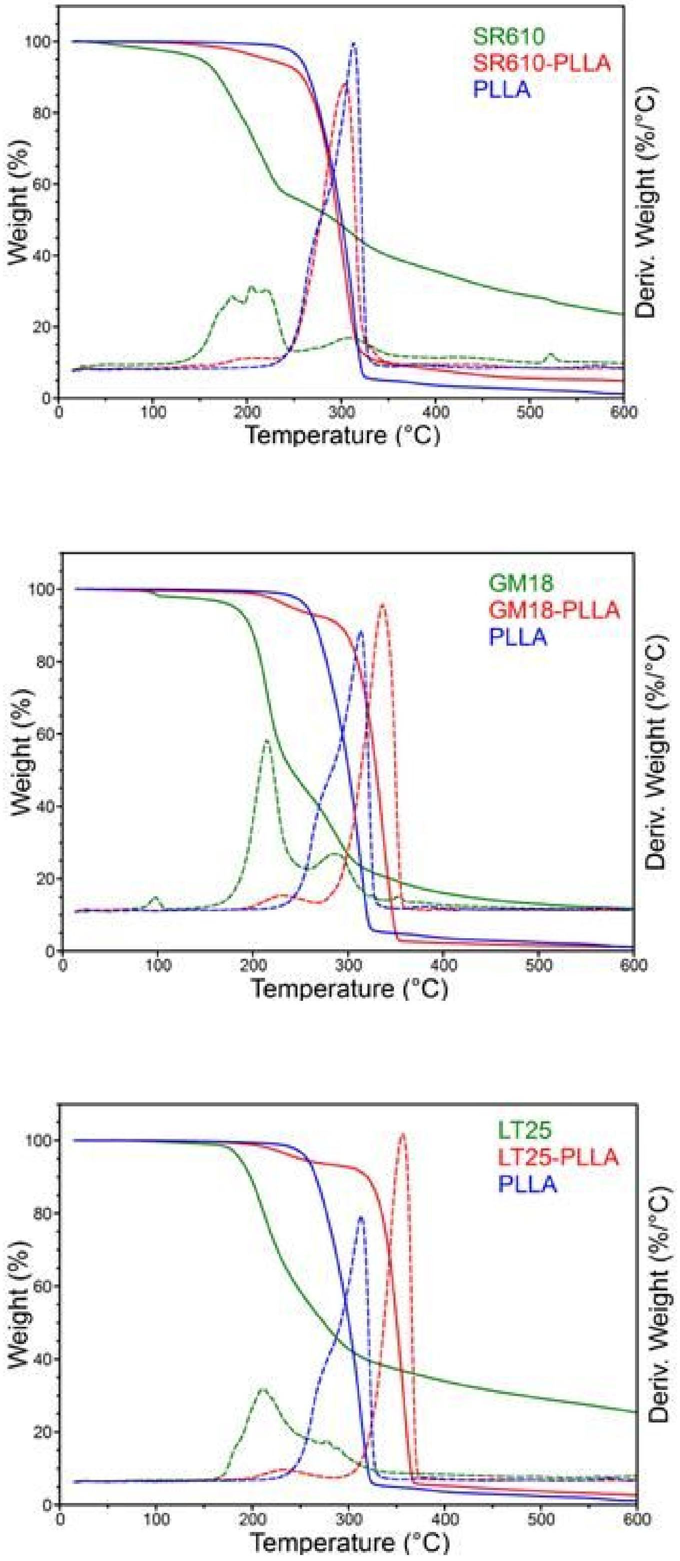

Figure S3. Overlay of thermogravimetric curves of the PLLA fibers functionalized with each compound (SR610, GM18, LT25**)**, the corresponding compound and plain PLLA (Solid line: weight %; dotted line: derivative weight %/°C)

DSC Results

The DSC curve in Figure S4 shows a glass transition at a temperature around 50-60 °C and a cold crystallization exothermic peak (T_c around 120 °C), followed by a melting endothermic peak (T_m around 155 °C) of the same entity ($\Delta H_c = \Delta H_m$ around 35 J/g). This result indicates that the melting phenomena that follows the cold crystallization concern only the PLLA crystal phase developed during the heating scan, thus demonstrating that completely amorphous fibers were obtained.

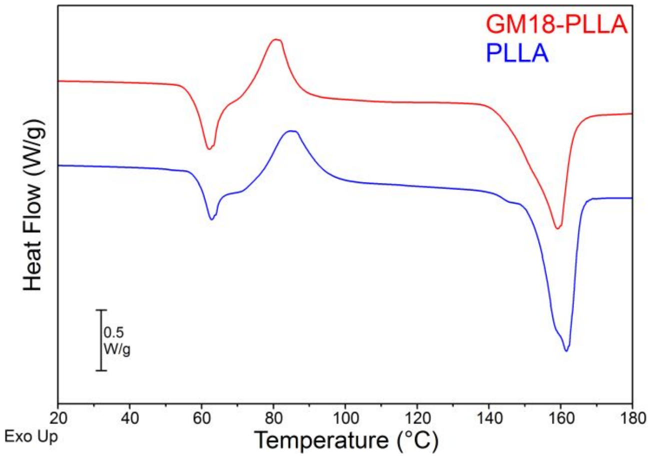

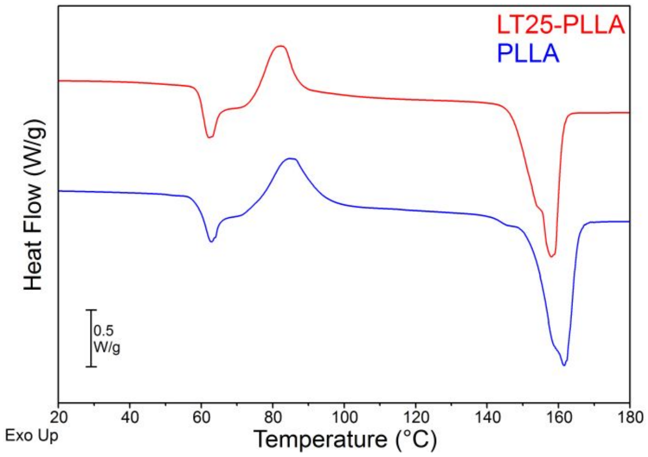

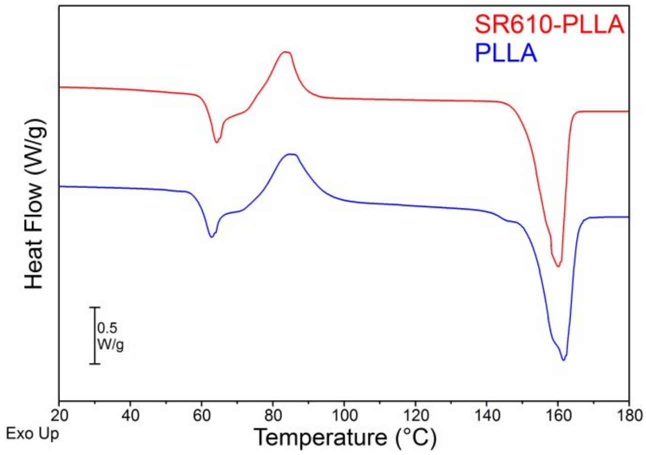

Figure S4. Differential Scanning Calorimetry (DSC) analysis of samples of SR610-, GM18-, LT25-PLLA, and plain PLLA (first heating scan, 20°C/min).

Table S2: Thermal properties of PLLA and β-lactam-containing PLLA

Sample	T^a_g (°C)	$\Delta C^a p$ (J/g°C)	T^b_c (°C)	ΔH^b_c (J/g)	T^b_m (°C)	ΔH^b_m (J/g)
PLLA	60	0.65	125	38	161	38
**GM18**-PLLA	50	0.57	122	30	155	30
**LT25**-PLLA	56	0.50	122	34	156	35
**SR610**-PLLA	57	0.50	110	37	15	38

a: heating scan (20°C/min) after quench from melting; b: first heating scan (20°C/min)

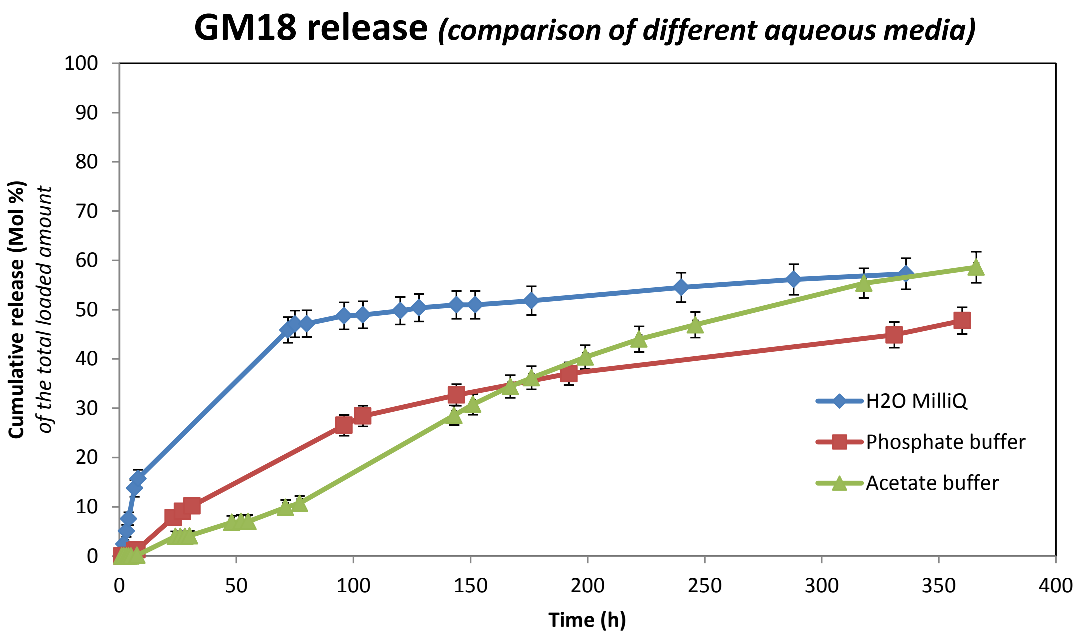

Figure S5. Release of β-lactam **GM18** in aqueous solution MilliQ-H_2O(♦ blue), phosphate buffer solution at pH 7.4 (▪ red), acetate buffer solution at pH 5.0 (▴ green). Data were obtained in triplicate. Bars represent the mean values ± SD (standard deviation of the means) of results from three experiments. The scaffolds were used as such for the release studies. The cumulative release was reported as mol % over time (hours).

	**SR610**-PLLA	**GM18**-PLLA	**LT25**-PLLA
Dry scaffolds			
Wet scaffolds			

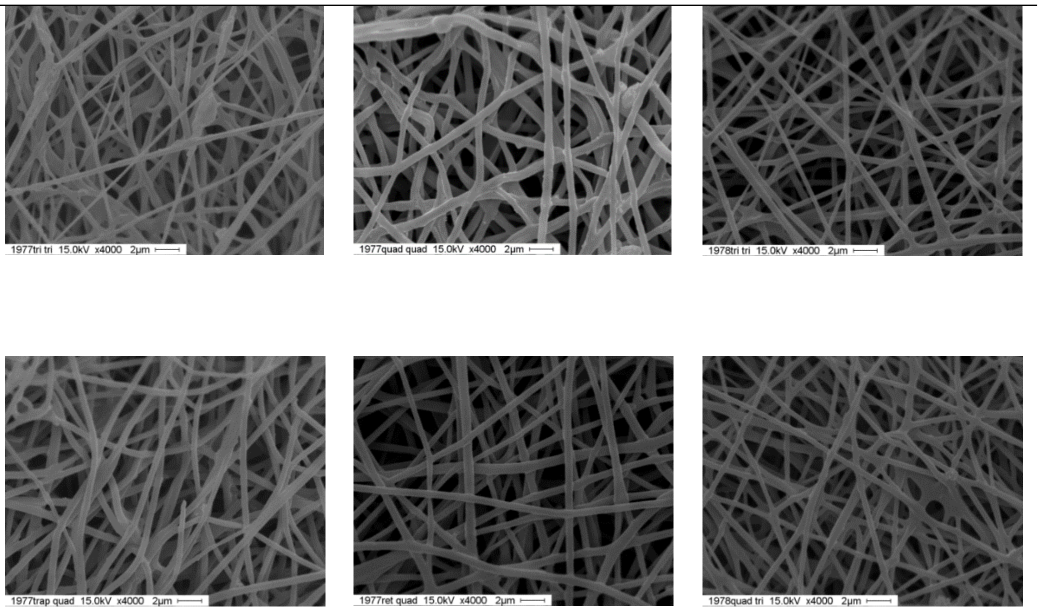

Figure S6. SEM images (4000×) of SR610-, GM18-, LT25-PLLA samples after release study in dry and pre-wetted treatment.

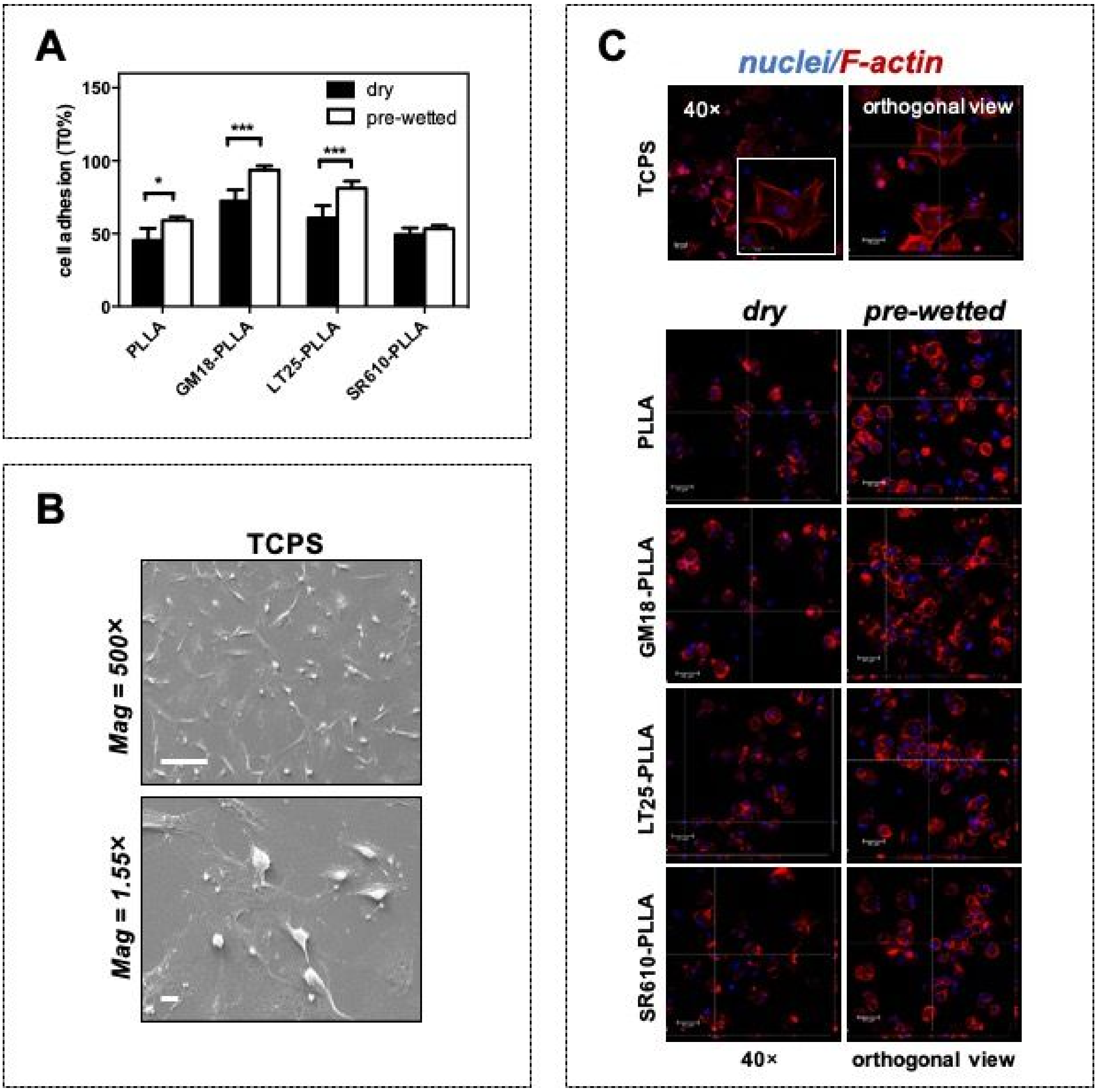

Figure S7. Cell adhesion of hBM-MSCs cultured on plain PLLA and agonist-PLLA scaffolds. Cell adhesion was plotted as percentage of viable cells in comparison with initial seeded cell number (T0). A) The differences in cell adhesion between dry and pre-wetted conditions (*** $p < 0.001$; * $p < 0.05$) were shown. Bars represent the mean values ± SD of results from three experiments ($n = 3$). B) Cell morphology was assessed by scanning electron microscopy (SEM). Representative SEM images of the cells cultured on the TCPS (magnification 500× and scale bar: 100 µm; mag 1.5× and scale bar: 10 µm). C) Cell morphology was also assessed by confocal laser microscopy analysis of hBM-MSCs. Images of cells seeded onto TCPS (20 and 40× magnification), PLLA and the different agonist-PLLA scaffolds (20×) were reported. The cytoskeleton organization was observed by F-actin staining with Phalloidin (red), whereas nuclei were stained with Hoechst 33342 (blue). After 2 hours from seeding, cells cultured on TCPS exhibited a well spread shape, associated with a dense meshwork of defined F-actin stress fibers

distributed throughout the cell-body. Scale bars: 50 µm. Orthogonal view of images stacks are also shown-visible.

Evaluation of the biological activity of unloaded GM18, LT25 and SR610 agonists

The biological activity of the three unloaded different agonists (GM18, LT25 and SR610) was assessed against human hBM-MSCs as described by Galletti P. and colleagues [*Galletti P, Soldati R, Pori M, Durso M, Tolomelli A, Gentilucci L, Dattoli SD, Baiula M, Spampinato S, Giacomini D. Targeting integrins αvβ3 and α5β1 with new β-lactam derivatives. Eur J Med Chem. 2014 Aug 18;83:284-93. doi: 10.1016/j.ejmech.2014.06.041.].* In brief, in the first set of experiments the ability of GM18, LT25 and SR610 to modulate the adhesion of hBM-MSCs to immobilized fibronectin (10 µg/mL) was evaluated (**Figure S8**). Interestingly, β-lactams GM18, LT25 and SR610 showed a concentration-dependent enhancement in fibronectin-mediated adhesion of hBMSC cells. The most potent agonist in enhancing cell adhesion with an EC50 of 3 nM was GM18 (**Figure S8**).

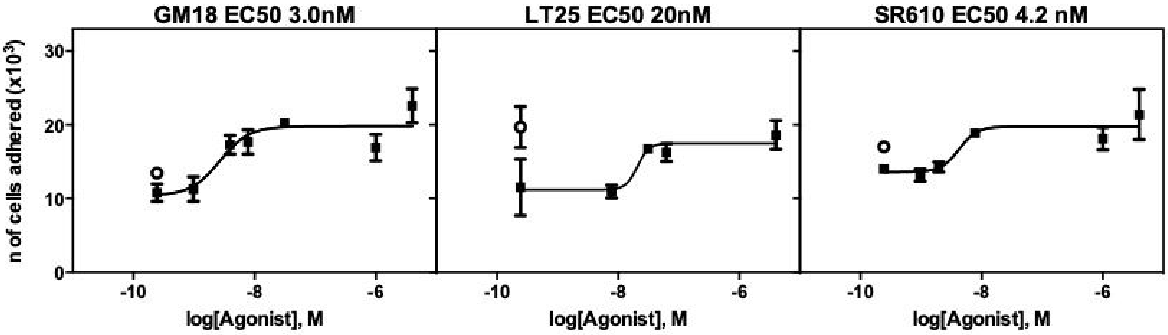

Figure S8. Compounds GM18, LT25 and SR610 enhance fibronectin-mediated adhesion to hBM-MSCs. Concentration-response curves showing the effects the GM18, LT25 and SR610 compounds on hBM-MSCs adhesion are reported. Cells were incubated for 30 min at room temperature with each compound (■) or with the vehicle (○). After 2 hours, cell viability was evaluated by MTT assay. Results were expressed as cell numbers attached to fibronectin-coated plates (±SD, n = 3).

In a second set of experiments, the ability of the new β-lactams to increase cell adhesion was then tested in absence of fibronectin. The adhesion of cells to wells previously coated by passive adsorption of all compounds, fibronectin and BSA (positive and negative control, respectively), was analyzed. After 2 h incubation, the cells adhered in all compound-coated wells, though some differences among them were observed: a higher cell adhesion was obtained in GM18 and SR610, comparable to fibronectin coating (**Figure 9A**). No cells adhesion to wells coated with bovine serum albumin alone was detected. Moreover, as evidenced by immunofluorescence analysis (**Figure S9B**), after 2 h culture on immobilized

GM18 and SR610, hBM-MSCs were able to spread across the surfaces extending their processes and developing F-actin fibers similarly to those seeded onto fibronectin. Additionally, the immuno-localization of pFAK-Tyr^{397} by immunostaining with specific antibodies highlights its cytosolic and membrane distribution, thus demonstrating the establishment of strong functional adhesion of cells to these compounds (**Figure S9B**). Conversely, a delayed process in adhesion activation was observed in cells incubated onto LT25, as clearly evidenced by an impaired cytoskeleton architecture as well as a cytosolic distribution of pFAK (**Figure S9B**).

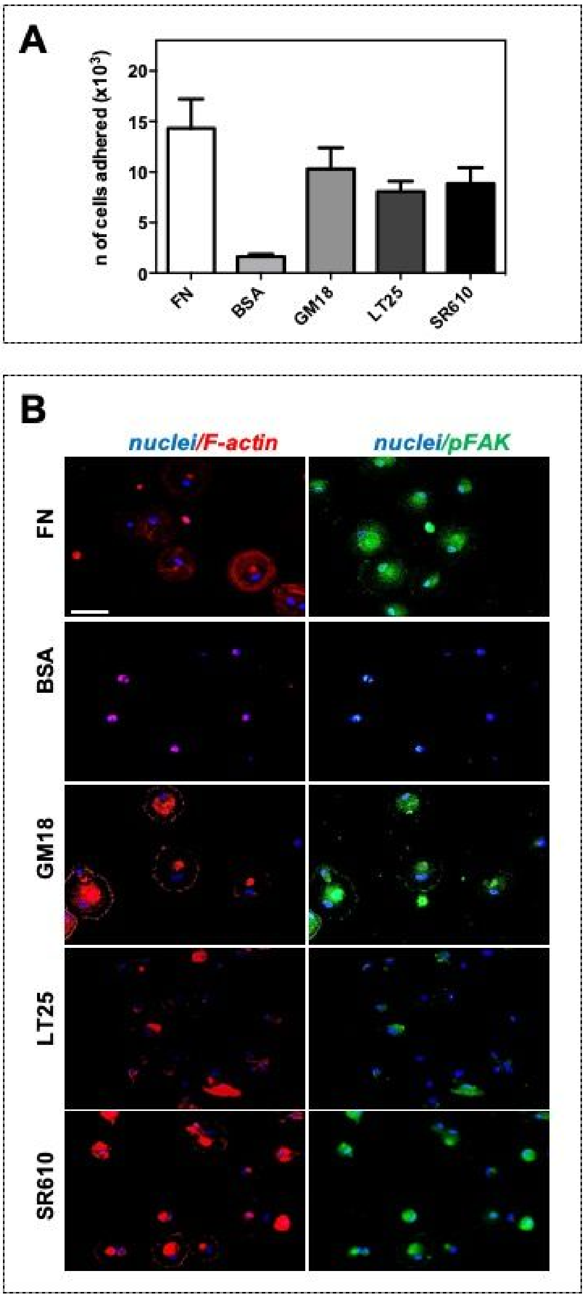

Figure S9. A) hBM-MSCs cell adhesion to plate wells coated with 10 ug/mL of fibronectin (FN) (positive control), and with different compounds. Cells plated onto wells coated with 10 ug/mL bovine serum albumin (BSA) represented the negative control. After 2 h, cell viability was evaluated by MTT assay. Each value was indicated as the mean ± SD (n = 3). B) CLSM observation of hBM-MSCs morphology after seeding onto coated plate wells with 10 ug/mL of FN, and with different compounds. The cytoskeleton organization was observed by F-actin staining with Phalloidin-TRITC (red). The expression of focal adhesion pFAK (green) was detected by a specific antibody. Nuclei were stained with Hoechst 33342 (blue). Scale bar: 100 µm.

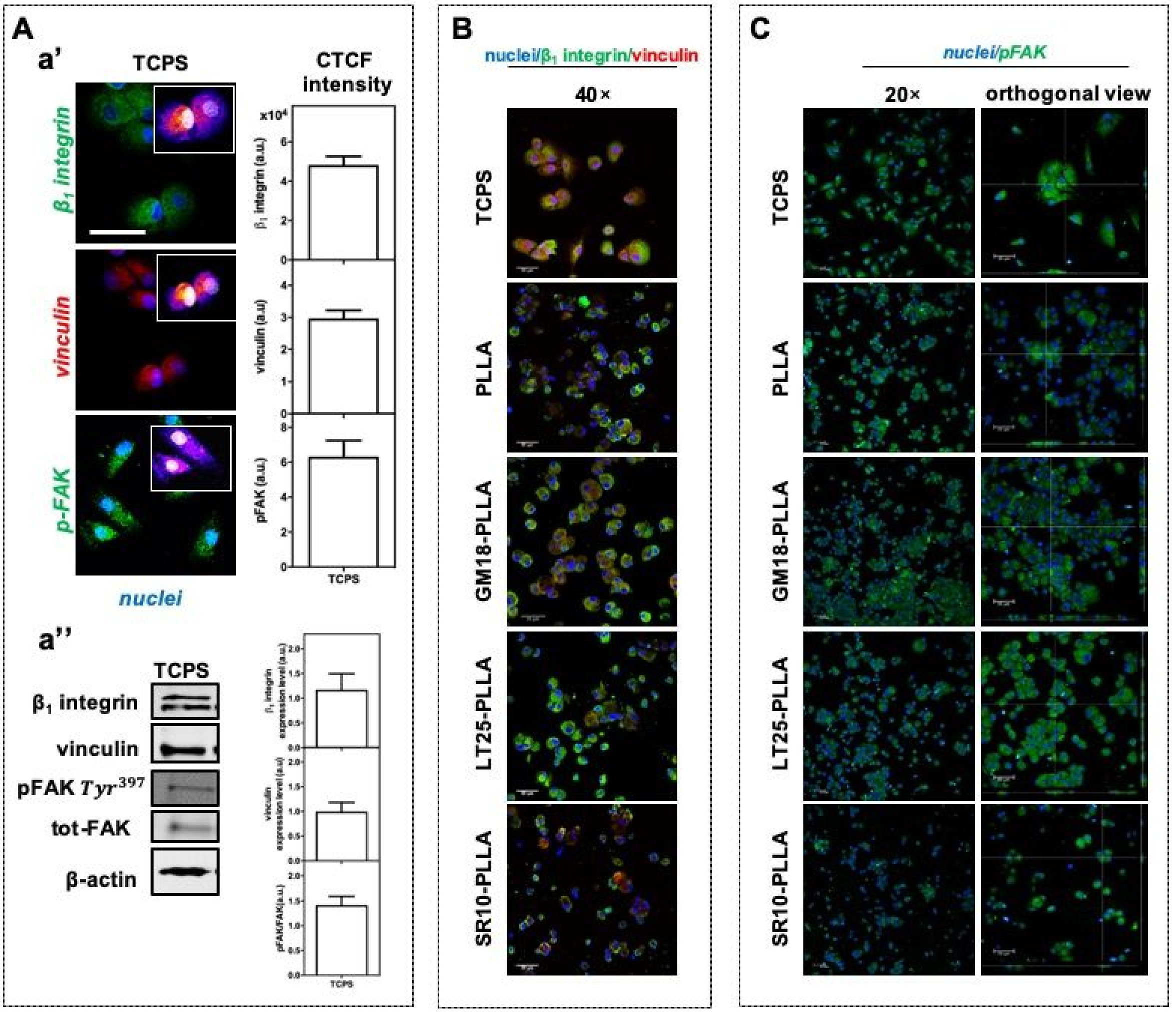

Figure S10. Qualitative and quantitative analysis of integrin, vinculin and pFAK after 2 h from seeding onto TCPS, plain PLLA, and agonists-functionalized PLLA scaffolds. Aa') Immunofluorescence images of nuclei (blue, Hoechst 33342), β_1 integrin (green, 488 Alexa Fluor), and vinculin (red, 633 Alexa Fluor) of hBM-MSCs cultured onto TCPS and relative corrected fluorescence intensity. Aa'') Western blot analysis of indicated proteins from lysates obtained by hBM-MSCs grew onto TCPS. B) Immunofluorescence from cells cultured onto TCPS, plain-PLLA and agonists-PLLA scaffolds. Scale bars: 50 µm; 40× magnification. C) Images showing the expression of focal adhesion pFAK (green, 488 Alexa Fluor) and nuclei (blue, Hoechst 33342) were acquired at a magnification of 20×. pFAK orthogonal view of images (40×) are also shown-visible. Scale bars: 50 µm.

Table S3. Primer sequences used for real-time qPCR

target	Forward primer (5' - 3')	Reverse primer (5' - 3')	Amplicon size (bp)
ITGα4	AATGGATGAGACTTCAGCACT	CTCTTCTGTTTTCTTCTTGTAGG	278
ITGα5	GTGGCCTTCGGTTTACAGTC	AATAGCACTGCCTCAGGCTT	181
ITGαV	GATGGACCAATGAACTGCAC	TTGGCAGACAATCTTCAAGC	207
ITGβ1	GAGGAATACAGCCTGTGGGT	ATTGCAGGATTCAGGGTTTC	121
ITGβ5	GTATGCTGGTTTTACAGACTCC	TGCCCTTTTGTAGCCTCCTTG	407
GAPDH	AGCCTCAAGATCATCAGCAATGCC	GTGGTCATGAGTCCTTCCACGAT	120
